# Evolution of coding and non-coding genes in HOX clusters of a marsupial

**DOI:** 10.1186/1471-2164-13-251

**Published:** 2012-06-18

**Authors:** Hongshi Yu, James Lindsay, Zhi-Ping Feng, Stephen Frankenberg, Yanqiu Hu, Dawn Carone, Geoff Shaw, Andrew J Pask, Rachel O’Neill, Anthony T Papenfuss, Marilyn B Renfree

**Affiliations:** 1 ARC Centre of Excellence in Kangaroo Genomics, The University of Melbourne, Melbourne, Victoria, 3010, Australia; 2 Department of Zoology, The University of Melbourne, Melbourne, Victoria, 3010, Australia; 3 Department of Molecular and Cell Biology, College of Liberal Arts and Sciences, University of Connecticut, Connecticut, CT, 06269, USA; 4 Bioinformatics Division, The Walter and Eliza Hall Institute of Medical Research, Parkville, VIC, 3052, Australia; 5 Department of Medical Biology, The University of Melbourne, Melbourne, Victoria, 3010, Australia; 6 Department of Mathematics and Statistics, The University of Melbourne, Melbourne, Victoria, 3010, Australia

**Keywords:** Marsupial, *HOX* cluster, MicroRNAs, Long non-coding RNAs

## Abstract

**Background:**

The HOX gene clusters are thought to be highly conserved amongst mammals and other vertebrates, but the long non-coding RNAs have only been studied in detail in human and mouse. The sequencing of the kangaroo genome provides an opportunity to use comparative analyses to compare the HOX clusters of a mammal with a distinct body plan to those of other mammals.

**Results:**

Here we report a comparative analysis of *HOX* gene clusters between an Australian marsupial of the kangaroo family and the eutherians. There was a strikingly high level of conservation of *HOX* gene sequence and structure and non-protein coding genes including the microRNAs *miR-196a*, *miR-196b*, *miR-10a* and *miR-10b* and the long non-coding RNAs *HOTAIR*, *HOTAIRM1* and *HOX*A11AS that play critical roles in regulating gene expression and controlling development. By microRNA deep sequencing and comparative genomic analyses, two conserved microRNAs (*miR-10a* and *miR-10b*) were identified and one new candidate microRNA with typical hairpin precursor structure that is expressed in both fibroblasts and testes was found. The prediction of microRNA target analysis showed that several known microRNA targets, such as *miR-10*, *miR-414* and *miR-464*, were found in the tammar *HOX* clusters. In addition, several novel and putative miRNAs were identified that originated from elsewhere in the tammar genome and that target the tammar *HOXB* and *HOXD* clusters.

**Conclusions:**

This study confirms that the emergence of known long non-coding RNAs in the HOX clusters clearly predate the marsupial-eutherian divergence 160 Ma ago. It also identified a new potentially functional microRNA as well as conserved miRNAs. These non-coding RNAs may participate in the regulation of *HOX* genes to influence the body plan of this marsupial.

## Background

The origin, evolution, function and regulation of *HOX* genes are amongst the most intriguing questions in developmental biology and evolutionary genetics. Their highly conserved clustered arrangement on chromosomes, their spatio-temporal expression and their patterning results in each distinctive body plan during embryogenesis and organogenesis in bilaterian animals [1,2]. *HOX* genes are expressed as early as the pre-somite stage of gastrulation in the posterior primitive streak of the epiblast, a region that gives rise mainly to the lateral plate and extraembryonic mesoderm in chicken and mouse embryos [3-5]. The dynamic expression of *HOX* genes in the ectoderm, mesoderm and endoderm during gastrulation suggests that *HOX* genes are key regulators of regional patterning along the antero-posterior (A-P) axis [2-4,6]. *HOX* genes confer positional information for proper organ development and are expressed in ordered patterns that control the segmentation of the hindbrain and axial skeleton along the A-P axis, while mis-expression or mutation leads to the conversion of one structure into another, (homeotic transformation) [2]. Limb development and regeneration depends on patterning formation along three axes: A-P, dorsal ventral (D-V), and proximal distal (P-D) axes [7], where *HOX* A and *HOX* D, especially groups 9–13, are responsible for positional information along the A-P and P-D axes [8,9]. De-regulation of the *HOX* network results in cancers including breast, bladder, prostate and kidney, as well as abnormal expression during proliferation, differentiation and apoptosis and signal transduction [1,10].

In all vertebrates, *HOX* genes are comprised of two exons, in which exon 2 includes the highly conserved 180 bp of homeobox region, and a variable length of intron, from less than 200 bp to several kilobase pairs. The homeodomain encoded by a homeobox consists of 60 highly conserved amino acids and forms an N-terminal extended structure followed by three alpha helices. The homeodomain binds target DNA sequences at its N-terminal arm and the third helix from the minor and major groove of DNA, respectively. Orthologues of every *HOX* gene, including the homeodomain and flanking regions, are highly conserved among species. However, within species, the most conserved region between paralogues is restricted to the homeodomain. *HOX* genes are clustered on different chromosomes and are believed to have evolved from a single ancestral *HOX* gene by tandem duplications and sequence divergence [1,11]. There are four *HOX* clusters, denoted A, B, C and D, produced by two successive whole genome duplication events followed by subsequent divergence [12,13]. Paralogues within each cluster are designated 13 to 1 based on gene 5′-3′ transcribing orientation although there are only 11 paralogues at most found so far in vertebrates.

The low density of interspersed repeats in the human *HOX* clusters suggests that cis-regulatory elements are important in the tight control of *HOX* gene expression [14]. Global enhancer sequences located outside the clusters regulate *HOX*D temporal co-linearity [15]. Non-coding RNAs known to be involved in regulation of *HOX* gene expression [16,17], include the highly conserved microRNAs [18], such as *miR-196*[19] and *miR-10*[20]. The long non-coding RNAs *HOTAIR*[21,22] and *HOTAIRM1*[23] are known only in the mouse and human.

The comparison of *HOX* genes between vertebrates and invertebrates has highlighted conserved features of *HOX* gene expression regulation and evolution. Comparisons of DNA sequences between evolutionarily distantly-related genomes are highly efficient ways to identify conserved (and novel) functional regions, especially non-coding RNAs, and to discover how they regulate *HOX* gene expression [24,25]. However, some conserved functional features show lineage-specific distributions and will be missed if the taxa chosen are too distant in evolutionary terms. Similarly, if they are too close, differences can be missed. Marsupials fill the mammalian “gap” because they are a distinct lineage that diverged from eutherian mammals 130–160 Ma ago [26-29], but they are still mammals. There is a high ratio of conservation signal to random noise in comparisons between therian mammal (marsupial and eutherian) genomes, suggesting that there are localized regions under evolutionary constraint [30]. The divergence time between these groups is sufficient for non-functional sequences to have diverged while important genes are sufficiently conserved to enable their clear identification. Comparative genomics between eutherians and marsupials is therefore invaluable for predicting new and novel mammalian-specific motifs participating in *HOX* gene expression and regulation during mammalian evolution.

In this study, we used the tammar wallaby (*Macropus eugenii*), a macropodid marsupial of the kangaroo family, as our model. We screened BAC clones and further characterized all 39 tammar *HOX* genes as well as genome mapping and deep sequencing. Comparative genomic analyses identified the known *HOX* coding genes and non-coding regulatory regions including regulatory elements and non-coding RNAs. Importantly, we uncovered a new potential microRNA in the tammar HOX cluster.

## Results

### Sequencing and assembly

**Figure 1:**
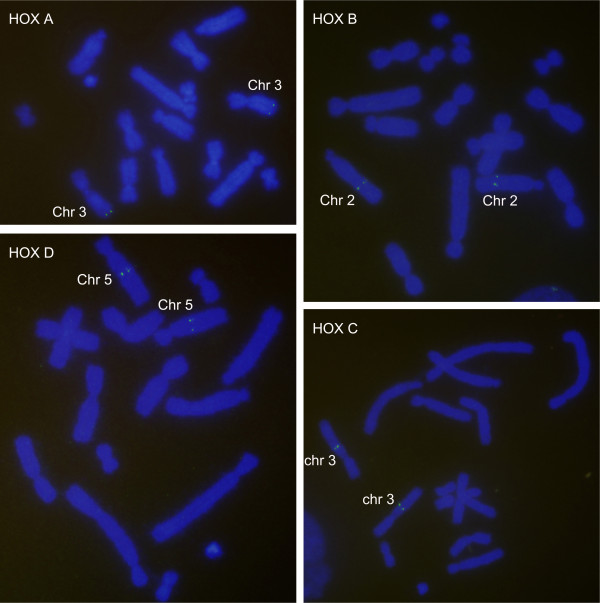
**Chromosomal locations of tammar*HOX*genes by Fluorescence*In-Situ*Hybridization.** Tammar *HOX* genes were mapped to four different chromosomal loci. BAC DNA was hybridized to metaphase chromosomes from a male donor stained with DAPI (blue). The hybridization signal was indicated with anti-DIG-FITC (bright green). *HOX*A was on the long arm terminal region of chromosome 3; *HOX*B was located 2/3 of the distance from the centromere on the long arm of chromosome 2; *HOX*C was on the middle of long arm at chromosome 3; *HOX*D was on the middle of long arm at chromosome 5.

To map *HOX* clusters on the tammar chromosomes (Figure 1), partial sequences of 34 tammar *HOX* genes were retrieved using the assembled tammar genome (assembly 1.0) [31] and the trace archives in GenBank. The *HOX* genes and clusters were highly fragmented in the genome assembly, so we used these sequences to screen a BAC library (Me_KBa; Arizona Genomics Institute, Tucson, AZ, USA) and then utilized a shotgun sequencing approach. Five BAC clones covering the *HOXA* to *HOXD* clusters were pulled down, sequenced using the Roche 454 platform and *de novo* assembled (Genbank: JN378718, JN378719, JN378720 and JN378721). Contigs were aligned to the genomic sequences of *HOX* clusters from opossum (Oct. 2006, MonDom5), platypus (Mar. 2007, WUGSC5.0.1/ornAna1) and human (Feb. 2009, GRCh37/hg19). These alignments verified that there were 37 *HOX* genes contained in the five BAC clones (see Methods), with *HOXA1* and *HOXD13* missing. Therefore, cross-species primers were designed to obtain full-length sequences for *HOXA1* and *HOXD13*. In addition, to confirm that *HOXA1* and other *HOXA* genes were clustered together, we screened a different tammar BAC DNA library (MEB1 library constructed at RIKEN, Japan). The newly obtained clones containing *HOXA1* also included *HOX*A genes identified by PCR. The same strategy was also used to confirm that *HOXD13* was clustered with other *HOXD* genes.

### Annotation of *HOX* clusters

**Figure 2:**
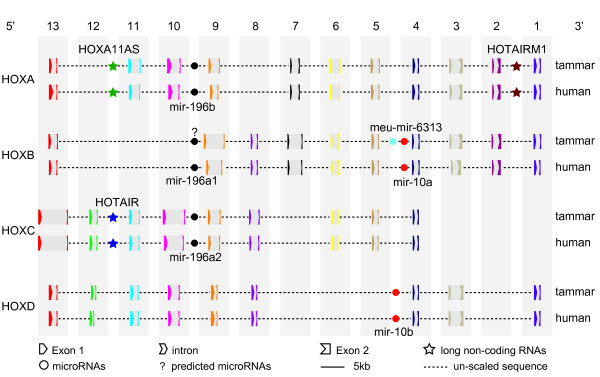
**Organization of*HOX*gene clusters, long non-coding RNAs and microRNAs in human and tammar.** The tammar has 39 *HOX* genes located in 4 separate clusters—*HOX*-A, -B, -C and -D—which show highly conserved organization. Three conserved long non-coding RNAs (*HOXA11AS*, *HOTAIRM1* and *HOTAIR*) were also present. Orthologous genes are the same color and introns filled with the grey color. The homologous long non-coding RNAs are the same color in the star while homologous microRNAs are also the same color. Question mark (?) represents the predicted microRNAs by sequence alignment.

Tammar *HOX* genes were clustered at four different loci with an arrangement of *HOX13* to −*1* from 5′ to 3′ (Figures 1, 2), showing a similar and highly conserved relative order and orientation of *HOX* genes in each cluster. There are 11 *HOXA* genes in the *HOXA* cluster, 10 *HOXB* genes in the *HOXB* cluster, 9 *HOXC* genes in the *HOXC* cluster and 9 *HOXD* genes in the *HOXD* cluster, showing a conserved distribution of homologues across each *HOX* cluster. Gene sequences have been submitted to Genbank with the BAC sequences. Tammar *HOX* genes each consist of two exons (detailed in Additional files 1, 2) encoding a highly conserved homeodomain, as is found in other vertebrates, including humans. Each *HOX* gene showed a similar and highly conserved overall exon length as well as sequence. Although the intron of each *HOX* orthologue varied significantly in sequence, the length of each intron is also conserved (Additional file 1), suggesting a high level of conservation across *HOX* clusters during evolution.

The abundance of repetitive DNA elements is extremely low in the core of tammar *HOX* clusters, in agreement with the previous findings in gnathostome *HOX* clusters [32]. Utilizing RepeatMasker (http://www.repeatmasker.org/cgi-bin/WEBRepeatMasker), repeat elements including short interspersed repeat elements (SINEs), long interspersed repeat elements (LINEs), long terminal repeats (LTRs) and other DNA elements were investigated in each tammar *HOX* cluster (Additional file 3). Strikingly, there were no Alu (short interspersed repeat element of about 300 bp, comprising 10.75% of the human genome), ERVL (long terminal repeats), TcMar-Tigger and satellite sequences found in any tammar *HOX* locus, resembling the human *HOX* clusters [14].

### Tammar HOX gene expression in adult tissues

**Figure 3:**
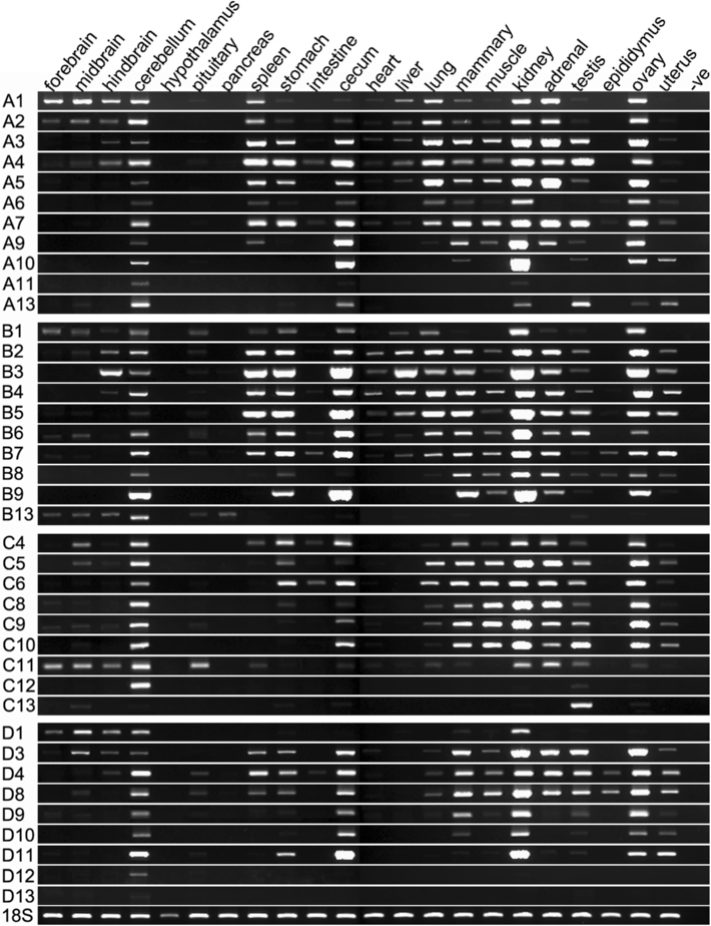
**Tammar*HOX*gene expression in adults.** Tammar *HOX* gene expression pattern were examined in 23 adult tissues including brain, cerebellum, hypothalamus, pituitary, pancreas, spleen, stomach, intestine, cecum, heart, liver, lung, mammary gland, skeletal muscle, kidney, adrenal, testis, epididymis, ovary, uterus and prostate with 39 *HOX* genes primers (Additional file 12). A, *HOX*A; B, *HOX*B; C, *HOX*C; D, *HOX*D; 18S, housekeeping gene and positive control.

The expression patterns of all 39 HOX genes were analysed in 23 adult tissues by RT-PCR, including brain, gastrointestinal tract, circulatory system, digestive system and reproductive system of the tammar wallaby (Figure 3).

Some anterior HOX genes (*HOX1* to −*3*) were expressed in the forebrain, midbrain and hindbrain in tammar, similar to the expression patterns of human HOXA genes [33], but very few HOX genes were expressed in hypothalamus, pituitary and pancreas. Interestingly, almost all HOX genes were expressed in cerebellum, suggesting that HOX genes continue to participate in coordinating motor activity and communication as they do during development [34,35]. Anterior (1–3) and central (4–8) HOX genes of cluster A/B/D were expressed in the spleen and carry important roles in replenishing red blood cells and in activating the immune response. In the tammar gastrointestinal tract, weak expression was found in intestine while much stronger expression was observed in stomach and caecum, showing tissue-specific expression patterns. Anterior and central HOX genes of clusters A and B, but not C or D, were expressed in liver and heart. In tammar lung tissue, almost no posterior HOX genes were expressed. Skeletal muscle had broad expression of HOX genes (HOX1-11). HOX gene expression in reproductive tissues was similar to those in the developing tissues, displaying ongoing proliferation, differentiation, and degeneration of multiple cell types. HOX genes were strongly expressed in the mammary gland, kidney, adrenal, testis and ovary, but had a restricted expression in epididymis and uterus. Overall, HOX genes had tissue-specific expression patterns, maintaining high expression in some tissues, while in other tissues they were down-regulated or switched off.

### Functional and conserved non-coding sequences in the kangaroo *HOX* clusters

**Figure 4:**
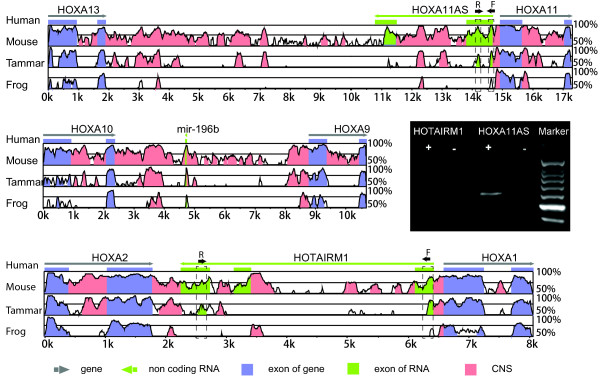
**Conserved miRNA and long non-coding RNAs analysis in the*HOX*A cluster.** The conserved long non-coding RNAs, *HOXA11AS* and *HOTAIRM1*, and microRNA *miR-196b* were shown by mVISTA with comparison of mouse, tammar and frog against human *HOX*A cluster genomic sequence. The coding genes *HOXA13*, *HOXA10*, *HOXA9*, *HOXA2* and *HOXA1* are highly conserved in all species. Expression of tammar long non-coding RNAs in bone marrow and endometrium were confirmed by RT-PCR. The blue stands for coding regions, and the green for non-coding RNA regions whilst the pink represents conserved coding sequences. F, forward primer, R, reverse primer.

**Figure 5:**
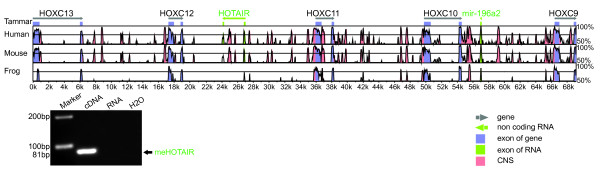
**Sequence conservation in the*HOX*C cluster in tammar, human, mouse and frog.** mVISTA plot of *HOX*C genomic sequences from tammar, human (chr12:54332691–54396455), mouse (chr15:102751619–102814560) and frog (scaffold_226:281324–390491). The sequence similiarity (50–100%) (vertical axis) is shown in the coordinates of the genomic sequence (horizontal axis) from human, mouse and frog. Genes and their orientation are indicated by grey arrowed line. Exons of genes are indicated by blue solid boxes. Conserved regions above the level of 70%/100 bp are highlighted under the curve, with red indicating conserved non-coding regions, blue representing conserved coding-protein exons, and turquoise representing microRNAs or long non-coding protein exons. The long non-coding RNA *HOTAIR* located between *HOXC12* and *HOXC11* was conserved in all mammals and had a much lower conservation in frog. MicroRNA *miR-196a2* is extremely highly conserved in all examined species. RT-PCR performed in the tammar with a single band at 81 bp confirmed the presence of the long non-coding RNA *HOTAIR* providing further evidence of the conservation. In addition, both microRNA *miR-196a2* was expressed in tammar cells, verifying the existence of this microRNAs in tammar

Comparative genomic analysis between tammar, human, mouse and a non-mammalian vertebrate, frog (Additional files 4, 5, 6, 7) using mVISTA [36], showed that the coding regions of each cluster were highly conserved, whereas the non-coding regions including untranslated regions (UTRs), intergenic regions and introns shared a comparatively low sequence similarity but were conserved in length. Furthermore, there was higher conservation in the 3′ UTR of each *HOX* gene than in the 5′ UTR, similar to previous findings [24] (Figures 2, 4, 5 and Additional files 4, 5, 6, 7). This provided a platform to identify whether these conserved non-coding sequences function as conserved transcription factor binding sites or non-coding RNAs participating in gene expression regulation/RNA processing, or whether they just act as non-functional and randomly conserved elements, maintaining high sequence identity for about 500 Ma of evolution for vertebrates or up to 160 Ma of evolution for mammals [26,28].

### Known long non-coding RNAs are conserved in the kangaroo *HOX* clusters

Long non-coding RNAs (lncRNAs) play critical roles in transcription regulation, epigenetic gene regulation and diseases. They are rapidly evolving genes, and are expected to be poorly conserved at the sequence level [37-39]. However, we found conserved orthologues of all three known mammalian lncRNAs—*HOTAIRM1HOXA11AS* and *HOTAIR* (sequences provided in Additional file 8)—by comparative genomic analysis and RT-PCR amplification.

**Figure 6:**
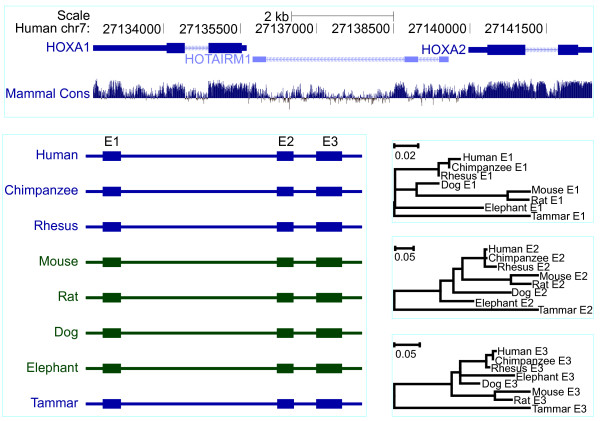
**Comparative genomic analysis of*HOTAIRM1*orthologues in mammals.** The genes flanking *HOTAIRM1*, *HOXA1* and *HOXA2,* from the human genome (chr7:27,132,617–27,142,393; http://genome.ucsc.edu), are shown along with their conservation score (phylop). *HOTAIRM1* gene structure consists of three exons in eutherian mammals, but two exons in the tammar (lower left), based on predicted RNA secondary structure and sequence alignment. Phylogenetic trees showing that exon 1 is highly conserved with short genetic distance between them compared to exon 2 and exon 3 consistent with the concept of rapid evolution of non-coding RNAs (lower right).

*HOX* antisense intergenic RNA myeloid 1 (*HOTAIRM1*) was located between *HOXA1* and *HOXA2*, and we demonstrated that it was restricted to mammals (Figures 4, 6 and Additional file 4). The tammar *HOTAIRM1* has three exons according to RT-PCR size. Exon 1 was highly conserved across all mammals. Exon 2 could not be detected in tammar and opossum using the “Infernal” (v1.0.2) program (http://infernal.janelia.org/), which employs both RNA secondary structure and sequence to search the genomic sequence, but using RT-PCR, we were able to find exon 2. The conservation of the secondary structure of exon 3 is lower than that of exon1, but is much higher than that of exon 2, which can be clearly observed by the phylogenetic trees in the right bottom panels (Figure 6). *HOTAIRM1* was expressed in bone marrow as expected (Figure 4), suggesting that this lncRNA has had conserved roles in myelopoiesis across all mammals for up to 160 Ma.

**Figure 7:**
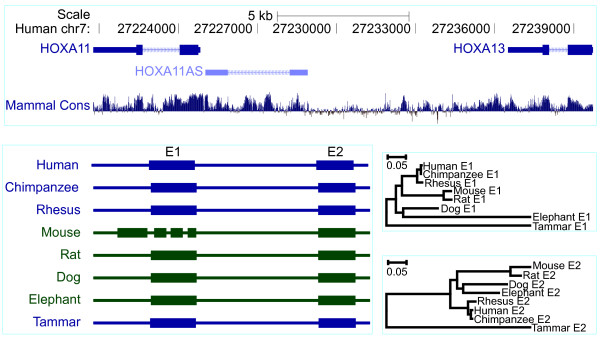
**Evolutionary relationships of*HOX*A11AS orthologues. **The genes flanking *HOXA11AS*, *HOXA11* and *HOXA13*, in the human (chr7:27,220,777–27,239,725; http://genome.ucsc.edu) are shown along with their conservation score (phylop). *HOXA11AS* gene structure consists of two exons in eutherian mammals except mouse, but one exon in tammar (lower left), based on predicted with RNA secondary structure and sequence alignment. Phylogenetic trees showing exon2 is highly conserved in eutherian mammals whilst exon1 is more divergent with the full predicted exon 1 sequence, consistent with the mammalian consensus sequences in the top panel (lower right).

Tammar *HOXA11* antisense (*HOXA11AS*), located between *HOXA13* and *HOXA11*, has two exons similar to that in human (Figures 4, 7 and Additional file 4). *HOXA11AS* was highly conserved in eutherian mammals, but had a very low conservation in marsupial species, while in the frog it had less than 50% identity. Additionally, using the “Infernal” program we found that exon 1 had a conserved RNA secondary structure, but it failed to predict exon 2. Although there was a low conservation between tammar and eutherian mammals, tammar *HOXA11AS* was expressed in the endometrium during pregnancy (Figure 4) as it is in humans, suggesting a conserved role in mammalian reproduction.

**Figure 8:**
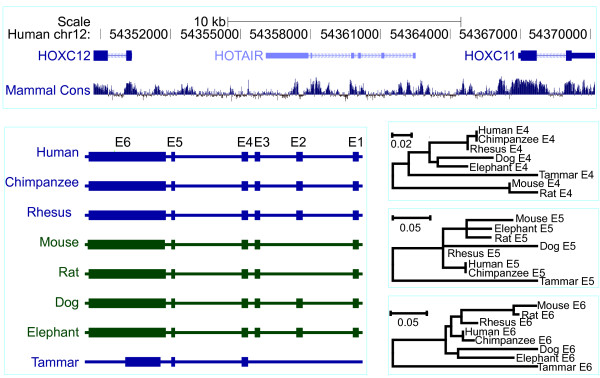
**Evolutionary relationships of HOTAIR orthologues.** The genes flanking *HOTAIR*, *HOXC11* and *HOXC12*, in the human genome (shr12:54,348,714–54,370,201; http://genome.ucsc.edu) are shown along with their conservation score (phylop). *HOTAIR* gene structure consists of 6 exons in the eutherian mammals, except mouse and rat, which have 5 exons. In contrast, only 3 exons were found in tammar. Phylogenetic trees based on exons 4–6 (lower right).

*HOX* antisense intergenic RNA (*HOTAIR)* is a trans-regulatory gene, unlike the other lncRNAs that are cis-regulatory. It plays an important role in epigenetics and tumorigenesis. In the tammar, it was located between *HOXC11* and *HOXC12* as in human and mouse. Exon 1–4 are very short exons of about 100 bp, and exon 5 is just 53 bp in human, but there was only low conservation seen in the mVISTA plot (Figure 8 and Additional file 6). Exon 6 is the longest exon, showing some regions that are highly conserved (Figures 5, 8 and Additional file 6). In the tammar, exon 1–3 could not be identified with the “Infernal” program using human RNA secondary structure of each exon to search the tammar *HOX* genomic sequence. However, exon 4 is highly conserved in all species and was easily identified using RNA secondary structure or sequence itself. The phylogenetic tree further confirmed this phenomenon, showing it had a much short genetic distance compared to the other exons (Figure 8). Although tammar exon 5 was identified using the “Infernal” program, it showed very low sequence conservation (only 53 bp) and a long branch length in the phylogenetic tree (Figure 8). Tammar exon 6 was much shorter than that of other species. Exon 6 was highly conserved in eutherian mammals (Figure 8) but the short tammar sequence was conserved with the equivalent sequence in eutherians. Tammar *HOTAIR* was present in a RT-PCR of a whole day 20 fetus at the early head-fold stage (Figure 5 and Additional file 6) and was also expressed in the developing limbs at least at day 23 of gestation [40] as in the human and mouse [21,22,41], suggesting that this lncRNA may be involved in trans-regulation of limb development in all mammals [40].

### The kangaroo *HOX* clusters encode conserved microRNAs

mVISTA plots showed numerous non-coding regions, possibly representing microRNAs, were highly conserved (Additional files 4, 5, 6, 7). We examined the presence of known microRNAs, *miR-196a1*, *miR-196a2*, *miR-196b*, *miR-10a* and *miR-10b*, previously described in the human, mouse and zebrafish HOX clusters. As expected, we found 5 known conserved miRNAs in tammar *HOX* clusters (summary in Figure 2 and the sequences provided in Additional file 8, genomic sequence alignment referred to Additional files 4, 5, 6, 7). We examined tammar microRNA deep sequencing libraries from different tissues and cells to determine the expression profile of each of these miRNAs. We found that *miR-10a* and *miR-10b* were strongly expressed in the testis. They are also expressed in fibroblast cells of the tammar.

**Figure 9:**
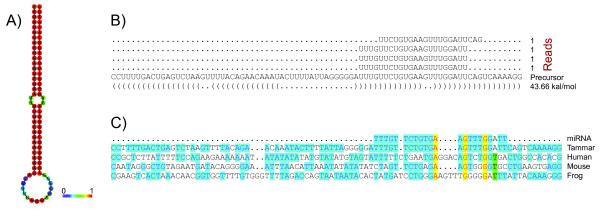
**Newly discovered miRNA *meu-miR-6313*in tammar. A**) Centroid secondary structure with a minimum free energy of −43.66 kcal/mol; the bar from blue to red represents base-pair probabilities from low (0) to high (1); **B**) the reads, precursor and secondary structure of new miRNA; **C**) sequence alignment of miRNA and precursors in tammar, human, mouse and frog.

In order to computationally explore new or novel miRNAs and their targets in the *HOX* cluster of the tammar wallaby, we developed special miRNA pipeline for tammar wallaby and programs (see Methods for details) by using our microRNA deep sequencing libraries, *HOX* cluster sequence obtained by our BAC sequencing, tammar whole genome sequence and miRBase (http://www.mirbase.org/). Interestingly, we found one new potentially functional miRNA with a distinct hairpin structure that is expressed in fibroblasts and testis (Figure 9). Regarding targets of miRNAs in the tammar HOX clusters, valid miRNA hits to *miR-10a*, *miR-10b*, *miR-414* and *miR-466* were confirmed (details referred to Additional file 9). Unfortunately, we could not find the target of our newly discovered candidate microRNA in HOX cluster. However, we found several new targets in HOX clusters that are novel putative microRNAs with hairpin structures but their exact location in the tammar genome could not yet be determined (Additional file 10).

## Discussion

Comparative genomic analysis of the marsupial *HOX* clusters uncovered a new microRNA and confirmed the presence of numerous known mammalian RNAs. There was a strikingly high level of conservation of coding sequences between this member of the kangaroo family and that of eutherian mammals.

Marsupial *HOX* gene clusters are compact and uninterrupted by large repeat domains. In the tammar, the length of all clusters were remarkably similar to that found in human (tammar *HOXA-D*: 113 kb, 207 kb, 144 kb and 110 kb; human *HOX*A-D 112 kb, 205 kb, 137 kb and 112 kb retrieved from the UCSC genome browser GRCh37/hg19). Similar patterns are also found in frog, chicken and mouse (Additional files 4, 5, 6, 7), demonstrating that the *HOX* gene clusters are highly conserved and compact across vertebrate lineages. However, *Amphioxus*, which is viewed as an “archetypal” genus in the chordate lineage, carries a *HOX* cluster length of about 448 kb [42]. In invertebrates, *HOX* clusters are often more than 1 Mb, as is found in the sea urchin [43]. Thus the vertebrate *HOX* clusters are more compact than the ancient and invertebrate *HOX* clusters [42].

All 39 tammar *HOX* genes had conserved gene structures (Additional file 11) and chromosomal arrangement (Figure 2), consistent with the theory that two rounds of genome duplications occurred after the vertebrate–invertebrate divergence but before bony fishes and tetrapods split [12,13,44]. In adults, *HOX* genes continue to be expressed and thereby retain developmental plasticity in certain tissues or maintain homeostasis. However, there has been much less work on gene expression in adult tissues compared to developing tissues [45,46]. We showed that *HOX* gene expression in adult marsupial tissues was tissue-specific and differentially expressed (Figure 3). Interestingly, almost all *HOX* genes were expressed in the cerebellum, suggesting that *HOX* genes continue to participate in coordinating motor activity and communication in adults, as they do during development [2].

Using the tammar *HOX* genomic sequences as a reference for phylogenetic footprinting, we were able to identify a large number of conserved non-coding genomic sequences which may act as transcription factor binding sites in promoters, regulatory motifs involved in chromatin remodeling or non-coding RNAs that modulate gene expression post-transcriptionally [25,47]. Long non-coding RNAs play diverse roles in biological processes but are thought to be under different evolutionary constraints and are expected to have low sequence conservation compared to protein-coding sequences [38], which has hampered the study of long non-coding RNA in vertebrates. We not only found these lncRNAs orthologues in the tammar *HOX* genome, but also confirmed that they were expressed in certain tissues. For example, human *HOTAIRM1* is expressed specifically in myeloid cells to regulate *HOXA1* and *HOXA4* expression in NB4 cells (an acute promyelocytic leukaemia cell line) [23]. Tammar *HOTAIRM1* was also expressed in bone marrow, suggesting it has a conserved role in myelopoiesis across all mammals. In addition, *HOTAIRM1* appears to be restricted to mammals and so must have evolved during the mammalian radiation. A recently discovered long non-coding RNA, *HOTAIR*[21,22], acts as a trans-regulator to regulate *HOX*D but not *HOX*C gene expression during limb development [22] and participates in reprogramming chromatin states to promote cancer metastasis [21]. Tammar *HOTAIR* was also found in the tammar *HOX* genomic sequence [31], and was expressed at the early head-fold stage of the tammar embryo at the time just before limb buds develop, suggesting that it may have a role in the regulation of limb development—especially important structures for the kangaroos [40]. In addition, the 5′ flanking sequence of *HOTAIR* was conserved, suggesting that it has the same or similar transcriptional regulation mechanism (Figure 5 and Additional file 6). Thus, contrary to expectation, mammalian lncRNAs do show a reasonable level of sequence conservation.

Micro-RNAs are highly conserved, in contrast to long non-coding RNAs, and play important roles in animal development by controlling translation or stability of mRNAs [48]. They are normally 22 nucleotide RNA that binds to complementary sequences in the 3′UTR to repress gene activities [49]. Using the tammar as a reference and searching the microRNA database we were able to identify four known *HOX* microRNAs (*miR-196amiR-196bmiR-10a* and *miR-10b*), and most significantly, we uncovered one new potential microRNA, *meu-miR-6313* in the tammar which was expressed in testis and fibroblasts. The precursor sequence was used to search the human, mouse, and frog genomes and was not present (Figure 9). We also searched the opossum and Tasmanian devil genome sequences using the precursor sequence plus of 1 kb flanking sequences. While the flanking sequences were conserved in these two other marsupial species, we did not find the sequence immediately around the precursor, suggesting that it is a recent insertion in tammar. *In silico* analysis as well *in vitro* and *in vivo* experiments have shown that the miRNAs *miR-10* and *miR-196* target several HOX genes, such as HOXA5/7/9, HOXB1/6/7/8, HOXC8, HOXD8, HOXA1/3/7, HOXB3 and HOXD10 [18-20,50,51]. In this study, we also predicted targets of miRNAs, and found the targets of *miR-10amiR-10bmiR-414* and *miR-466* in the HOX clusters (Additional file 9). We also found numerous new targets whose microRNAs precursor genes were located outside the HOX clusters in the tammar genome (Additional file 10). These novel microRNAs have a typical secondary hairpin structure and targets in the HOX clusters. These miRNAs may participate in *HOX* gene expression and regulation to control the kangaroo type body plan and hopping mode of locomotion. Thus, using the tammar *HOX* as the reference genome, the examination of the marsupial *HOX* gene clusters has uncovered new and known non-coding RNAs of mammals.

## Conclusions

Annotation and comparative genomic analysis of tammar *HOX* genes demonstrated a high degree of evolutionary conservation. As expected, 39 *HOX* marsupial genes were mapped to four different chromosomal loci. The tammar *HOX* clusters had a low concentration of repetitive elements and were compact as in other vertebrate *HOX* clusters. The protein-coding regions and their UTRs also showed high conservation but there was a novel potentially functional miRNA *meu-miR-6313* within a HOX cluster. Interestingly, the long-coding RNAs (*HOTAIR*, *HOTAIRM1* and *HOXA11AS*) and microRNAs (*miR-196a2*, *miR-196b*, *miR-10a* and *miR-10b*) were highly conserved in this marsupial. These lncRNAs and miRNAs may control the *HOX* genes to influence phenotypic differences in the body plan, as they do in other mammals. This study confirms that the emergence of known long non-coding RNAs in the HOX clusters clearly predates the marsupial-eutherian divergence up to 160 Ma ago.

## Methods

### Animals, tissues and cells

Tammar wallabies originating from Kangaroo Island, South Australia, were held in the University of Melbourne marsupial breeding colony in Melbourne, Victoria. All sampling techniques and collection of tissues conformed to Australian National Health and Medical Research Council (2004) guidelines and were approved by The University of Melbourne Animal Experimentation & Ethics Committees.

Tissues (forebrain, midbrain, hindbrain, cerebellum, hypothalamus, pituitary, pancreas, spleen, stomach, intestine, caecum, heart, liver, lung, muscle, kidney and adrenal) were collected from five adults. Bone marrow, mammary glands, uterus and ovary were collected from three adult females. Prostates, epididymides and testes were collected from two adult males. *HOX* gene expression was examined using all tissues listed above except bone marrow. Bone marrow, whole embryos (day 20 of the 26.5 day gestation, n = 2) and endometrium (collected from three additional pregnant females) were used to examine lncRNA expression. All tissues were collected under RNase-free conditions. All collected tissues for molecular analysis were snap frozen in liquid nitrogen and stored at −80°C until use.

Tammar primary cells were prepared from a day 10 post partum pouch young testis. Briefly, the primary cells were cultivated in 50% DMEM (containing 10% fetal bovine serum) (Invitrogen, Melbourne, Australia) and 50% AminoMax (Gibco, Carlsbad, USA) containing 15% fetal calf serum.

### Probe preparation and BAC library screening

The six frame translation of the tammar genome (assembly 1.0) was searched for homeobox domains using a profile hidden Markov model (Pfam accession PF00046.21) and the HMMer software (version 2.3.2) [52]. An E-value threshold of 10^−4^ was used. Predicted homeobox domain sequences of at least 80aa and related DNA were extracted from the tammar genome. The domain classes of these sequences were then classified using *HOX*Pred [53]. At the same time, tammar *HOX* partial sequences were also obtained by searching the tammar trace archives with human exon 1 and exon 2 of 39 *HOX* genes using BLASTN. Gene specific primers were designed to amplify probes and to confirm identity of isolated BACs. All primers and their annealing temperatures as well as the product size are listed in Additional file 12.

The tammar BAC library (Me_KBa) with average insert size of 166 kb was constructed by M. Luo at AGI (Me_KBa; Arizona Genomics Institute, Tucson, AZ, USA). Radioactively ^32^P-labelled PCR probes from 5′ and 3′ (*HOX*A to *HOX*D) were used to screen the BAC library. Resulting positive BACs for each *HOX* cluster were further confirmed with all corresponding *HOX* genes by PCR.

When screening the BAC library, at least two probes from the 5′ end and 3′ end were selected and 5 positive clones were identified: 205I5, 9G11, 168N24, 6P18 and 214D22. BAC clone 205I5 covered *HOX*A cluster genes (*HOXA2* to *HOXA13*); BAC clone 9G11 covered the *HOX*B cluster (*HOXB1* to *HOXB9*); BAC clone 168N24 covered the *HOX*B cluster (*HOXB4* to *HOXB13*); BAC clone 6P18 contained all *HOX*C cluster genes and clone 214D22 covered the *HOX*D cluster (*HOXD1* to *HOXD12*).

### BAC DNA preparation, sequencing and assembly

Positive BAC bacteria were cultured overnight in LB medium containing 12 μg/ml chloramphenicol at 37°C. BAC DNA was extracted according to manufacturer’s instructions of Maxipreps DNA purification system (Promega, Sydney, Australia). The quality was assessed by gel electrophoresis in 0.8% agarose gel and NanoDrop ND-1000 Spectrophotometer (Wilmington, USA) with the ratio of A260/A280 at over 1.8. The amount of DNA was also measured by NanoDrop ND-1000 Spectrophotometer. BAC samples were sequenced with GS-FLX method at the Australian Genome Research Facility Ltd (AGRF, Brisbane, Australia).

The Roche 454 reads of the tammar were extracted and *de novo* assembled with the program CAP3 [54]. There are 202 contigs from BAC 205I5 in HOXA cluster, 85 contigs from 168N24 and 2613 contigs from 9G11 in HOXB cluster, 405 contigs from 6P18 in HOXC cluster and 89 contigs from 214D22 in HOXD cluster. The contigs were then aligned to the genomic sequence of human, tammar, opossum and platypus and any gaps between the new contigs from the BAC sequencing filled where sequence was available using the tammar genome sequence. Based on these genomic sequences, gene structures of all *HOX* genes and full *HOX* scaffolds were identified.

### microRNA sequencing and *in silico* analysis

The recently published marsupial genome paper provided deep sequencing information [31] and additional sequencing of the tammar microRNAs was performed on an Illumina GAII platform. Briefly, 40 μg Trizol extracted total RNA from tammar brain, liver, testis, and pouch young fibroblast cells grown in culture was electrophoresed on a 15% denaturing polyacrylamide gel with γ-[32P]-ATP end labeled 19-mer, 24-mer and 33-mer oligonucleotides. The bands corresponding to the miRNA fraction (19–24nt) were excised and ligated to an adenylated 3′ adapter (IDT, Inc.). The 3′ ligated RNA was electrophoresed on a 15% polyacrylamide gel and the bands corresponding to miRNA were excised. A 5′ ligation reaction and subsequent polyacrylamide gel purification followed by reverse transcription and PCR was performed in preparation for Illumina sequencing. Sequencing was performed on an Illumina GAII according to the manufacturer’s protocol.

miRNAs mapped to HOX genome were performed using Bowtie [55], allowing for at most 1 mismatch. Potential hairpin locations were first identified using the SRNALOOP program (http://arep.med.harvard.edu/miRNA/pgmlicense.html). They were further refined by manual inspection of the hairpin loop using an interactive instance of RNAfold program (http://rna.tbi.univie.ac.at/cgi-bin/RNAfold.cgi). Target prediction was done using the miRanda tool [56] with default parameters. The novel microRNAs and the complete *HOX* genes were used as the query and target sequences, respectively.

### Phylogenetic footprinting analyses

For interspecies DNA sequence comparison, tammar or human genomic sequence acted as a reference in four species (Human, Mouse, Tammar and Frog). Genomic sequences containing *HOX*A, *HOX*B, *HOX*C and *HOX*D clusters from Human (*HOX*A, chr7: 27098056–27210689; *HOX*B, chr17: 43960868–44165742; *HOX*C, chr12: 52605461–52742874; *HOX*D, chr2: 176656359–176768195; released in Feb 2009), Mouse (*HOX*A, chr6: 52104079–52216539; *HOX*B, chr11: 96024912–96229585; *HOX*C, chr15: 102757899–102892969; *HOX*D, chr2: 74497085–74613489; released in July 2007) and Frog (*Xenopus tropicalis*) (*HOX*A, scaffold_56: 1381000–1485000; *HOX*B, scaffold_334: 483000–620000; *HOX*C, scaffold_226: 269568–557892; *HOX*D, scaffold_163: 534804–660354; released in Aug. 2005) were retrieved from UCSC website (http://genome.ucsc.edu/).

Alignment of each *HOX* cluster from these species and tammar were performed using the LAGAN algorithm available on the mVISTA website with default parameters [36]. The sequence from tammar was set as reference. The conserved tammar microRNAs were found in *HOX* genomic sequences by alignment of human/mouse microRNAs and further confirmed by deep sequencing and miRNA mapping [31]. Tammar specific and new conserved microRNAs were identified by deep sequencing and miRNA mapping [31]. Annotation of tammar long non-coding RNAs (lincRNAs) was performed according to human/mouse lincRNAs and confirmed by RT-PCR (primers in Additional file 12).

### RT-PCR

RNAs were isolated from various tissues with TRI Reagent solution (Ambion, Scoresby, Australia) following the instructions. The quality and integrity of the RNA was assessed by gel electrophoresis in 1% agarose gel and the quantity was measured with NanoDrop ND-1000 Spectrophotometer (Wilmington, USA). Total RNA was digested and purified with DNA-free™ DNase (Ambion, Scoresby, Australia) to remove the contaminated genomic DNA prior to cDNA synthesis. To ensure that there was no genomic DNA contamination, the quality of RNAs was accessed by PCR with primers in one exon.

Approximately 2 μg of total RNA was used as template for reverse transcription with the SuperScript III First-Strand Synthesis System for RT-PCR (Invitrogen, Melbourne, Australia) each reaction, using 1 μl of Oligo(dT)_20_ (50 μM). The quality of the first strand synthesis reaction was examined by PCR amplification of 18S standards.

About 20 ng of cDNA was used as a template for gene amplification with *HOX* genes specific primers (All sequences and annealing temperatures of primers are listed in Additional file 12). PCR cycling conditions were: 35 cycles of 30 s, 95°C; 30 s, 47–62°C; 30 s, 72°C, in a 25 μl reaction with GoTaq Green Master Mix (Promega, Sydney, Australia) and 0.4 μM of both forward and reverse primers.

### Comparative analysis of long non-coding RNAs

To perform comparative analyses of long non-coding RNAs, the following human genomic sequences were employed to outline sequence similarity and evo-lution in UCSC genome browser (http://genome.ucsc.edu/), *HOX*C12-HOTAIR-*HOX*C11 (Chr12: 54,348,714–54,370,201), *HOX*A1-HOTAIRM1-*HOX*A2 (chr7: 27,132,617–27,142,393) and *HOX*A13-*HOX*A11AS-*HOX*A11 (chr7: 27,220,777–27,239,725).

To search for the long non-coding RNAs, we retrieved the genomic sequences upstream to the nearest *HOX* gene and the corresponding downstream *HOX* gene in multiple eutherian mammals including chimpanzee, rhesus, mouse, rat, dog and elephant. The “Infernal” program (http://infernal.janelia.org/) was employed to blast each genome sequence with default parameters. Briefly, we used the secondary RNA structure of each exon in human lncRNAs to produce *.sto file. The secondary structure was predicted by online program RNAfold WebServer (http://rna.tbi.univie.ac.at/cgi-bin/RNAfold.cgi). Cmsearch of “Infernal” program was then used to build a model from above secondary structure. Cmcalibrate of “Infernal” program was used to determine expectation value scores (E-values) for more sensitive searches and appropriate HMM filter score cutoffs for faster searches. Cmsearch was used to blast genomic sequences downloaded from NCBI or Ensembl. Using cmsearch, the lowest E-value with less than 0.01 has the priority.

### Phylogenetic trees

A phylogenetic trees (Figures 6,7,8) of lincRNAs were constructed with MEGA 5.05 program [57]. Briefly, MUSCLE protocol was used to align DNA sequence from single corresponding exon of predicted lincRNAs and known exons in humans. When constructing trees, a maximum likelihood strategy was employed with default parameters.

Based on HoxPred [58], homeodomain regions plus 20 amino acids adjacent to their upstream and downstream region are enough to classify Hox proteins in their groups of homology. We therefore chose these sequences to perform phylogenetic analysis of HOX genes (Figure 3). The sequences were aligned with MUSCLE [59], and a neighbor-joining tree was built with JTT distance and bootstrap analyses by using the SeaView package [60].

### miRNA pipeline, miRNA and hairpin annotation

In order to computationally explore the cause and effects of miRNA in the HOX cluster of the tammar wallaby we followed a processes inspired by [61]. Our miRNA has three main goals; separating valid sequences from noise and degradation product, identifying miRNA targets and genes. The targets and genes of our pipeline can then be compared against known features from miRBase (http://www.mirbase.org/) to determine which are confirmed and which are novel.

Each sequenced library is pre-processed to remove both 3′ and 5′ prime adapters and is then size selected to remove reads with less than 15 or more than 32 bases. Next the reads were aligned against the HOX cluster allowing for no mismatches, all valid alignments for each read were reported. The same reads are aligned against the genome, except one mismatch is allowed to compensate for the draft nature of the tammar genome.

To separate between valid miRNA and degraded product/sequencing noise it is required that each read must align at least once within an annotated miRNA gene or hairpin region. The construction of this annotation is detailed in a later section. The novel miRNA gene in HOX was identified by during the annotation stage detailed in a later section. The novel miRNA targets required to meet the following conditions: 1) a valid read aligned to the HOX cluster, 2) the location of the aligned read did not overlap with a previously annotated target.

The main requirement of the miRNA pipeline previously presented is that each read must have aligned within an annotated miRNA gene or hairpin at least once in the genome. The miRNA gene annotations generally come from an external gene annotation pipeline such as ENSEMBL (http://asia.ensembl.org/info/docs/genebuild/genome_annotation.html). Since the tammar genome is quite new, and highly fragmented this annotation is incomplete. To augment it, the hairpin sequences in miRBase [62] are aligned to the genome using BLAST. The locations where the known hairpins align are considered equivalent to a miRNA gene.

To capture novel miRNA genes and hairpins, a simple pipeline of commonly available tools was created. Many published tools which identify new micro RNA genes use sequence and structure based alignments to find the best candidates [63]. Unfortunately these tools do not scale well and are too slow to use on large genomes and large micro RNA datasets. Therefore we implemented a custom version of the strategy mentioned;above. First, all miRNAs were mapped to the genome. Next, each aligned sequence plus 100 bp flanking windows were put into SRNALOOP a hairpin prediction tool [64]. Regions containing valid hairpins which did not overlap with a previously known miRNA gene or miRBase annotation were recorded.

### miRNA target annotation

miRNA targets were annotated in a two-step process. First the valid miRNA were mapped against the HOX cluster allowing for no mismatches. Then the mature miRNA from miRBase release 18 were mapped against the HOX cluster, allowing for 1 mismatch. A target was considered confirmed if a valid miRNA from our pool co-located with a miRNA from miRBase. Otherwise the aligned sequence was considered to be novel.

Our definition of a valid miRNA required each sequence to be associated with at least one miRNA gene, or hairpin structure somewhere in the genome. All of the putative novel miRNA targets in HOX were associated with a hairpin [table XYZ]. However, none of these hairpins were found within an annotated gene. This could be due to a poor annotation, the draft status of the genome, or it is simply a false signal. Each of these will be further validated in future research.

## Abbreviations

A-P, Antero-posterior; D-V, Dorsal ventral; ERVL, Endogenous retrovirus L; HOTAIR, *HOX* antisense intergenic RNA; HOTAIRM1, *HOX* antisense intergenic RNA myeloid 1; *HOX*A11AS, *HOX*A11 antisense; LINEs, Long interspersed repeat elements; lncRNAs, Long non-coding RNAs; LTRs, Long terminal repeats; MaLR, Mammalian LTR; MIR, Mammalian-wide interspersed repeats; P-D, Proximal distal; RTE, Retrotransposable element; SINE, Short interspersed repeat elements; UTRs, Untranslated regions.

## Competing interests

The authors declare that they have no competing interests.

## Authors’ contributions

The author(s) have made the following declarations about their contributions: conceived the study and designed the experiments: MBR, HY. Collected the tissues: HY, GS, MBR. Performed the experiments: HY, DC, YH, JL. Analysed the data: HY, JL, Z-P F, MBR, RO’N, AJP, ATP, SF. Wrote the paper: HY, MBR, AJP, ATP. All authors edited and approved of the manuscript.

## Authors information

Anthony T Papenfuss and Marilyn B Renfree are joint senior authors

## Supplementary Material

Additional file 1 **The length of exon and intron of tammar 39*HOX*genes (bp).** Click here for file

Additional file 2 **The sequences of 39 tammar*HOX* genes.** Click here for file

Additional file 3 **Repetitive elements in tammar*HOX*clusters.** Click here for file

Additional file 4 **Phylogenetic footprinting analyses of*HOX*A cluster with mVISTA.** mVISTA plot generated with *HOX*A genomic sequences from tammar, human (chr7:27131531–27244164), mouse (chr6:52104079–52216539) and frog (scaffold_56:1381000–1485000) with tammar as a reference. Conserved regions above the level of 70%/100 bp are highlighted under the curve, with red indicating conserved non-coding regions, blue representing conserved coding-protein exons, and turquoise representing microRNAs or long non-coding protein exons. *HOTAIRM1* and *HOXA11AS* representing the long non-coding RNAs are conserved in all mammals and have much lower similarity in frog. microRNA *miR-196b* is highly conserved in all species. Arrow stands for the transcription orientation. Click here for file

Additional file 5 **Phylogenetic footprinting analyses of*HOX*B cluster with mVISTA.** mVISTA plot generated with *HOX*B genomic sequences from tammar, human (chr17: 43960868–44165742), mouse (chr11: 96024912–96229585) and frog (scaffold_334: 483000–620000) with tammar as a reference. microRNAs *miR-10a* located between *HOX*B4 and *HOX*B5 is highly conserved in all species. *miR-196a1* is also conserved in all mammals. Other details as in figure Additional file 4. Click here for file

Additional file 6 **Phylogenetic footprinting analyses of*HOX*C cluster with mVISTA.** mVISTA plot generated with *HOX*C genomic sequences from tammar, human (chr12: 52605461–52742874), mouse (chr15: chr15:102750000–102892969) and frog (scaffold_226: 269568–557892) with tammar as a reference. The information of long ncRNAs and microRNAs is same as in Figure 3. Other details as in figure Additional file 4. Click here for file

Additional file 7 **Phylogenetic footprinting analyses of*HOX*D cluster with mVISTA.** mVISTA plot generated with *HOX*D genomic sequences from tammar, human (chr2: 176656359–176768195), mouse (chr2: 74497085–74613489) and frog (scaffold_163: 534804–660354) with tammar as a reference. Other details as in figure Additional file 4. Click here for file

Additional file 8 The sequences of lncRNAs and microRNAs in tammar. Click here for file

Additional file 9 **Valid target positions of known miRNAs in tammar *HOX*clusters.** Click here for file

Additional file 10 **Newly discovered target positions of putative miRNAs in tammar*HOX*clusters.** Click here for file

Additional file 11 **Phylogenetic relationships and high-order grouping of*HOX* families from tammar and human.** A representative an unrooted tree with rooting that should be considered arbitrary. Phylogenetic analysis was based on the homeodomain regions with an extension of extra 20 amino acids on both sides from human and tammar. The phylogenetic tree was constructed using neighbor-joining method with 100 bootstrap replicates showing bootstrap support values on the nodes. 13 monophyletic groups were shown to form *HOX*1 to *HOX*13. Three big branches according to their functions during the developmental events are shown: anterior, central and posterior. Click here for file

Additional file 12 Primers for RT-PCR. Click here for file
