# Supplementary material for: Evolution of coding and non-coding genes in HOX clusters of a marsupial
Source: BMC Genomics. 2012 Jun 18;13:251. doi: 10.1186/1471-2164-13-251 (PMC3541083; doi:10.1186/1471-2164-13-251)
Supplement: Additional file 1 — The length of exon and intron of tammar 39HOXgenes (bp). [file 1471-2164-13-251-S1.doc]

**Additional file 1: the length of exon and intron of tammar 39 HOX genes** (bp)

|  | **Exon1** | **Intron** | **Exon2** | **total** |  | **Exon1** | **Intron** | **Exon2** | **total** |
| --- | --- | --- | --- | --- | --- | --- | --- | --- | --- |
| ***HOXA1*** | 655 | 498 | 356 | 1509 | ***HOXC4*** | 439 | 501 | 350 | 1290 |
| ***HOXA2*** | 402 | 658 | 717 | 1777 | ***HOXC5*** | 454 | 743 | 212 | 1409 |
| ***HOXA3*** | 544 | 1568 | 770 | 2882 | ***HOXC6*** | 400 | 794 | 305 | 1499 |
| ***HOXA4*** | 562 | 459 | 323 | 1344 | ***HOXC8*** | 436 | 1503 | 290 | 2229 |
| ***HOXA5*** | 558 | 1141 | 249 | 1948 | ***HOXC9*** | 540 | 1905 | 240 | 2685 |
| ***HOXA6*** | 438 | 1602 | 264 | 2304 | ***HOXC10*** | 751 | 3692 | 275 | 4718 |
| ***HOXA7*** | 373 | 1349 | 308 | 2030 | ***HOXC11*** | 667 | 1381 | 230 | 2278 |
| ***HOXA9*** | 549 | 1602 | 234 | 2385 | ***HOXC12*** | 595 | 921 | 236 | 1752 |
| ***HOXA10*** | 976 | 1613 | 272 | 2861 | ***HOXC13*** | 766 | 5264 | 254 | 6284 |
| ***HOXA11*** | 719 | 1657 | 229 | 2605 | ***HOXD1*** | 670 | 406 | 332 | 1408 |
| ***HOXA13*** | 937 | 874 | 242 | 1732 | ***HOXD3*** | 541 | 1860 | 746 | 3147 |
| ***HOXB1*** | 565 | 745 | 329 | 1639 | ***HOXD4*** | 412 | 599 | 302 | 1313 |
| ***HOXB2*** | 388 | 952 | 704 | 2044 | ***HOXD8*** | 630 | 444 | 294 | 1368 |
| ***HOXB3*** | 444 | 1427 | 843 | 2714 | ***HOXD9*** | 774 | 369 | 237 | 1380 |
| ***HOXB4*** | 427 | 865 | 293 | 1585 | ***HOXD10*** | 744 | 1446 | 276 | 2466 |
| ***HOXB5*** | 555 | 760 | 246 | 1561 | ***HOXD11*** | 682 | 807 | 236 | 1725 |
| ***HOXB6*** | 412 | 1156 | 257 | 1825 | ***HOXD12*** | 565 | 297 | 236 | 1098 |
| ***HOXB7*** | 400 | 2538 | 251 | 3189 | ***HOXD13*** | 775 | 1083 | 248 | 2106 |
| ***HOXB8*** | 630 | 802 | 294 | 1531 |  |  |  |  |  |
| ***HOXB9*** | 510 | 3628 | 231 | 4369 |  |  |  |  |  |
| ***HOXB13*** | 601 | 1150 | 254 | 2005 |  |  |  |  |  |
