# Supplementary material for: Evolution of coding and non-coding genes in HOX clusters of a marsupial
Source: BMC Genomics. 2012 Jun 18;13:251. doi: 10.1186/1471-2164-13-251 (PMC3541083; doi:10.1186/1471-2164-13-251)
Supplement: Additional file 2 — The sequences of 39 tammarHOX genes. [file 1471-2164-13-251-S2.doc]

Additional file 2: The sequences of 39 tammar HOX genes (red, Exon1; black, Exon2)

>HOXA1

Atggacaatgcgagaatgagcgcctccttcctggagcaccctatcctcaacgcaggagactcggggacctgctccgcccgagcctaccaccccgatcatgggattacaactttccagtcgtgcgcggtcagtgccaacagctgcgggagcgacgagcgcttcctggtgggcaggggggtgcagatgagtcctccccaccaccctcacgcccatcaccactatccccagccggccgcctaccagcacctcaacaacctgggtatgtcttattcccactccagctgtggcccggggttcggatcgcagaacttcggagcaggttacagcccctactcattaaatcaggaagcagacgtaagcggcgggtacccctcgtgtgctcccgctgtttactctggaactctctcatcttccatggtccagcatcaccaccaccaccaccagggttatgctgggggcacggtggggtcgcctcagtacattcaccactcatatggacaagaagcgcagaatctgactctggcaaattataataactccttgtcccctctccatgccagccaccaagaaacctgtcgctctccttcagcagaggcatcacctccaacacagacctttgactggatgaaagtcaaaaggaatccccccaaaacagggaaagctggtgaatatgggtatattggccaacccaacacagtgcgcaccaattttaccaccaagcagctgactgagctggaaaaggaatttcatttcaacaagtacctcacgagggccagaagggtggagatcgcagcctcactgcaactcaacgagacccaggtgaaaatctggtttcagaaccgccgcatgaagcaaaagaagagagaaaaggagggcctgttgcccatttcgcccaccactccatcacggagtgacgacaaggccgaggaatcctcagacaaatctacctcttcgccctgcgtcccctcttccccagcctcctctacctcagacactctgactacctccaac

>HOXA2

Atgaattacgaatttgagcgagagactggctttatcaatagtcagccgtcgctcgctgagtgcctgacatcttttccccctgtcggtgatacatttcaaagttcatcaatcaagagctcgtcgctttcacagtcgacactgattcctcctccttttgagcagaccatccctagcctgaacccgggcagccaccctcgccacggcagcggcagcggcggcggccgccccaagtcgagtcctgatggcagcggcggcggtggtggcagcccggtgcccgctggcgccctgcagcccccggagtatccctggatgaaagagaaaaaagcttccaagaaaaccgcgctgcctcccgcctcctcggcctccgcctccgccactggacatgcctgcttgaaccacaaagaatccctggaaatcgcagataacagcagcgggggctccaggcgcctgagaacggcatacaccaatacccagctcttggagctagaaaaagaatttcatttcaacaagtatctctgtagacctcggagggtggagatcgcagccctgctggatttgactgagagacaagtgaaagtctggtttcagaaccgaaggatgaaacacaagaggcagacccagtgcaaagagaaccagcacagcgaggggaaatacaagaacctggaggacaccgagaaagccgaggacgaagaggagaaatcgctctttgagcaagcgctcaacgtctcaggggcacttctggagagggaagggtacacttttcagcaaaacgccctgtcccagcagcaggcccccaacgtgcacaatggcgactcccaaagtttcccagtttcgcctttaaccagcaatgagaaaaatctgaaacattttcagcatcagtcacccactgttcagaactgcttgtcaacaatgggcctgaacaatgacagcccagaggccttggatgtcccctctttacaagacttcaccgttttctccacagattcctgcctccagctttcagaggccgtatcgcccagtttaccaggctccctcgacagtcccgtagacatctcagctgacagctttgacttttttacagacacactcacgacaattgacttgcagcatctgaattac

>HOXA3

Atgcaaaaagcgacctactacgacagttcggcaatctacggcggatacccctaccaaggagcaaacggtttcacttataatgcgaatcatcagcaatatcctcagccctcggtgctggtggaaagcgaataccatcgtccagcctgctccctgcagtcccctgacagctctgtggcccgccacaaggccaatgacatcaacgagagttgcatgagaaccatcgggggccagggtggccaggctcagggccttcctgaatcccaacagcccccaccacagccccagcagcagcagcagctgcagcagcagcaggctccaccaccgtcgtcccaacagccccagcagccgtctcagccccctgttccttcggctccatcatcctcacagccccctccctctgtctcaccacctcaaaatgccagcagtaatccagcccccaccaagagtcccatgcttaattctcctaccatgtccaaacaaatctttccctggatgaaagagtctcgtcaaaacacaaagcagaaaaacagcagctccagttcaggtgagagttgctctggcgataaaagccccccggggcaggcatcatccaagagggctcgaacagcttacacaagtgcccagctggtggagctggagaaggaatttcacttcaatcggtatctgtgtagacctaggagggtcgagatggccaacttgctcaaccttaccgagagacagatcaaaatctggtttcagaaccgcaggatgaaatataaaaaggatcagaagggcaagggcatgatgacgtcctccggggggcagtcccccagtcgcagcccagttcctcctggcgctggggggtacctaaactctatgcattctctggtcaacagtgttccttacgagccgcagtcgcccccgtcctttagcaagcctcatcagaacacctatggcctccccaactcctaccctgctgcccttaacagctgcccaccgcctcagaagcggtactcggggactgcctcggtcactccagattacgaccctcaccccctgcaaggcaacggtagttatgggaccccacacttacagggaagccccgtcttcgtggggggcaactatgtggagcccatgagtaattccgggtcttccatctttggtctgactcatctctcccatcctgtctcagccagcatggactatggcggggctgggcccatagccaacaaccatcatcacggaccttgtgagccccaccccacctacacagaccttacagctcaccatccttctcagggaagaattcaggaggcgcccaaactgacccatctg

>HOXA4

Atgaccatgagctcgtttttgataaactccaactacctcgagcccaagttcccgccctgcgaggagttcggggcgcccggcggcccggacgggggaggctaccccccgcagcccccgccgcccccgccgccccagcacctgcagccgcccccgggggccgctcgctcgcccccgggctactaccgcccgccgcgacccgggccggagtcggggtacccggcgccggagtcggggtacccggcgcccccctccaccctctaccccgccccccccgccgccggcgcccgctaccagtgtggctacggccgcgccagccccggccggccccggcacgagcagcccccgcccccgccgcccccaacccaggggcctgcccgggggcctggggccggcccgagcctgctggccccgcagccgccgccggcgctcccccgccgcagcgaggctgccccggccgcgggcagccccccatggtgccccctgctcctggccgacaagagcctcaagggcggcaaggagcccgtggtgtacccctgtatgaaaaagatccatgtcagcaccgtgaaccccaattacaatggaggggaacccaaacgctcccgaaccgcctacaccaggcagcaggtcttggagctggagaaggagtttcactttaaccgttacctgacccggaggcgtcgcatcgagatcgctcacacgctctgcctctccgagcgccaggtcaagatctggttccaaaaccggaggatgaaatggaagaaggaccacaaactgcccaacaccaagatgcgctcggcggccccggccccgggcggccagccctgcaaagcacagactcagggcccgcatctgcccccgcgccaccagccgggtgcctccacgcta

>HOXA5

Atgagctcttattttgtaaactcattttgcggtcgctatccaaatggcccggactaccagttacataattatggagatcacagttccgtgagcgagccatacagggattcagcgagcatgcatcccagcaggtacggctatggttacaatggcatggatctcagcgttgggcgctcagcttccagccactttggtgccaatgagagagcccgcagttaccccgccaatgccacctcggcgtccacggagcccaggtataaccaacctgccacgtcgtctcactcgcctccacctgaccctctgccctgcaccgccgtggccgcttcgcctgtcagcgacagtcatcacggggtgaaaaactcgctagctaactccacgagcacatcgtccaattccagcagcactcacataagcagagagggggttggcacctcgtctgggactgaggatgacaccccagcgagcagcgagcaggcgagtgcccagagcgaccaaagtccagcccagccagcccaaccccagatctacccctggatgaggaagctgcatataagtcatgacaacataggaggaccagaaggcaaaagggctcggactgcatacacccgataccagaccctagagctggaaaaggaattccacttcaatagatacctgacccgcagaagaagaattgaaatagcacatgctctttgcctctcagagagacaaattaagatctggttccaaaacaggagaatgaaatggaaaaaagataataagcttaaaagtatgagtatggccgcggcgggaggggcttttcgcccc

>HOXA6

Atgagttcctattttgtgaatcccactttccctgggagcctgtctaatggacaggactccttcctcgggcagatccccctctatccagctggctatgatgctctgaggcatttccccgcttcttatggggcatcgagcctccaggacaagacatacacctcaccttgtttctaccaacagtccaacacggtcattgcttgcaatcgagcgtcctatgagtacggcacctcttgtttctactcagagaaggacctgagtagcgcctctccctcgggcagtggcaaacagagaggccacggggagtatctgcacttttctcctgagcaacaatacaaatccgaaagcagcgtgcagggcaaaatcttaaatgacgaaggtagcgaccggaagtacacgagtcctgtttatccctggatgcagcggatgaattcctgtgctggtactgtgtatggcacccacggccgaaggggaaggcaaacttacacgagatatcaaaccctcgaattggaaaaggaattccattttaaccgatacttgacgcgaaggcgccggattgagatcgccaacgcgctctgcctcacagaacgacagatcaaaatatggttccagaacaggagaatgaaatggaaaaaagagaacaaactcctaaattccacacagcccagcagcgaagaaacagaggaaaaggcagggggagtagaa

>HOXA7

Atgagttcttcgtattatgtgaatgcgctttttagcaaatatacggcgggggcttctctgttccaaaatgcagagccgacttcttgctcctttgccaccaactctcagagaagcggctacggaccgggcgcgggcgccttcgcttcctctgtgcccggcgtgtacaatgtgaacagtgccatttatccgggccccttctcgtccggatacggcctgggctcagacgcctacaacctgcactgctcgtcctttgatcagaatatccccgtcctctgcaatgacctaaccaaaggcagctgtgaaaaagccgaggcgagcgccctgcacagccaggcagagaccaatttccggatatacccttggatgaggtcttcaggtccggatcgcaagaggggtcgccagacctatacccgctatcagaccctggagctagagaaggagtttcatttcaaccgctatctgacccggcggcggcgcatcgagatcgcccacgccttgtgcctcaccgagaggcagatcaaaatctggttccagaaccggaggatgaagtggaaaaaggagcacaaggacgagggatctgctgccccggccgccaccgagagctcctcgaccaccccctcggcggggaaagtggaggaggaggaagatgaggaggaggaagacgaggaggaggaggaggaa

>HOXA9

Atgtcgacttcggggaccctgagcaattactacgtggactcgttcctgctccacgagagcgaagagctggtgccgtcccgctatggccccggcgctctggcgcagcccgccaggcagccggcgctgggggagcacccggacttcagcccctgcagcttccagtcgaaggcgtcggtcttcggcacttcctggaacccggtgcacccgcctaccgccaacaatgtgcccgccgtctaccacccctatgtgcaccaccaggcgccggtagcggcggcccctgacggcaggtacatgcgttcctggctggagcccatgccaggctctctgtccttcccgggtttaccttccagcaggcattacggcattaaacctgaaccgctctcggccagaaggggtgactgtaccacgtttgacactcacactttgtctctgtctgactatacttgtggttctcctccagctgatagggataagcagcccagcgaaggtgcgttcccggaaaacaatggagactgtgaggccggcggggacaaaccacccattgatccgaacccagctgcgaactggctacacgcgagatccacccggaaaaagcgctgtccctacaccaagcaccaaacgctagaactggagaaagaatttttgttcaatatgtatctcacccgggaccgcagatacgaggtggcaagactcctcaacctcactgaaagacaagtcaaaatatggtttcagaataggaggatgaaaatgaagaaaattaacaaagatcgagcaaaggacgaa

>HOXA11

Atggattttgatgagcgcgtgccctgctcgtctaacatgtatttgcctagttgtacttactacgtctcgggtcctgatttctcgagcctcccttcttttttgccccaaaccccgtcttctcgccccatgacatattcctattcgtccaacctcccccaggtacaacctgtaagagaagtgaccttccgggagtacgccattgagccctccagtaaatggcacccccgggccaacctgccccactgctattccgcagaggagctcatgcacagggaatgcctgcccgcctccggcagcgcgagcgtgggcgacatgctggccaagaactccgccaacgtctaccaccaccccagcaccacgggctcctcgtccaatttctatagcacggtgggcagaaatggggtcctgccccaggctttcgaccagtttttcgagactgcatacggccccccggacaacctgaccaccgcctcctcctctgactatccgggagataagggcgcggaaaaaatgcccccggcggccggggcggcggcggctgctgcagcagccccgccaacttcaagttcggatgccggcggcggaggctgcggccgggagaatgcggcggctgtggcggcggctgccgccgaggagaaggaaaggcgccggcggaccgaaagcagcagcagcccagagtcatcttccggcaacaatgaggacaaatccagcggctccagtggccagcggacccggaaaaaacgttgcccctacacaaaataccaaattcgggaactcgagagagaattcttcttcagtgtctatattaacaaggagaagcgtctccaactctctcgcatgctcaacctgaccgaccgccaagtcaaaatctggtttcagaacagacgaatgaaagaaaaaaaaattaacagagaccgattacagtactactctgccaacccgctactc

>HOXA13

atgacagcctccgtgctcctccacccccgctggatcgagcccaccgtcatgttcctctacgacaacggcggcggcctggtggcggacgagctcaacaagaacatggagggggcggcggcggctgctgctgctgcggcggcggcggccggggcggggggcgggggcttccaccaccctgccgccgcccacgcgggggggaacttctccgtggccgcggcggcggcggccgcggcggccgccgcgccaaaccagtgccggaacctgatggcgcacctggcgcccctgaccccgctctcagcgccccgggggtacagcgccccgggggaggcccccccatccgccgccgccgccgccgccgccgccgccgccgcagcgtcctcctcgtcgtcgtcctcgggcggcccgggacccgcgggggcggcgggcgcggagccggtcaagcagtgcagtccctgctcggcggcggcgcagagctcgtcgggcgccgccctgccctacggctacttcggcagcggttactacccgtgcgcccgcatgggccaccaccccaacgcgctcaagtcctgtgccgccgcggcgcagcccgcctcggccgccgccgccgctgccgccgccgccttcgctgacaagtacatggacacggctgcgggccccgcggcggcggcggccgaggagttcagctcccgcgccaaggagttcgccttctatcaccagggctacgcggcggggccctaccatcatcaccagcccgtgcccggctacctggatatgcccgtggtgccgggcctcgggggccccggtgagccccgccacgagccgctggggctgcccatggagagctaccagccctgggctttgcccaacggctggaatggccaggtgtactgccccaaagagcaaggccaacctccccacctctggaagtccactctgcccgatgttgtctcacatccttcggatgcgaactcctaccggaggggaaggaagaaacgagtgccttacaccaaggtccaattaaaagaactcgaaagggaatatgctacaaataaattcattactaaggacaaacggaggaggatatcagccacgacgaatctatccgagcgacaagtcaccatttggttccaaaacaggagggtcaaagagaaaaaagtcatcaacaaactcaagacgacaagt

>HOXB1

Atggactataataggatgaattccttcttggagtatgcactctgtaatcgaggtcctggagcctacagctcccctacatcttaccctccttgttcaagttctcccggtgacagctatggaggggaacctcgttatggaagtggtctccccagccctgtcctccagcagaatccaggctaccccacacagactccatcagcactgggggtccccttcaccaactcttcaccctcgggatacactgcacccacctgcagccctggctatggcccctcccaatactactccttgggccaacatgatggggatgggggctattttcagcagtcggggtatgggagccagctggggagcttatccgagacttatattacaggagggactggggcagggccctaccctccgccacagcccacttatgggactgagcaaccttcggccttcgggccatcctatggggatcttttagcggaggacaaggaaccaccctgcccgtcggagctgagcatgcccaccacccaaacctttgactggatgaaggtcaaacgaaaccctccaaagacagcaaaagtttcagatatgggcttgggcccagcgggtggcattcgcaccaacttcacaacgcggcagctgactgagctggagaaggaattccatttcaacaagtacttgagccgggcccgacgggtggaaattgccgccacactagagctcaatgagacgcaggtcaaaatttggttccagaaccggcggatgaagcagaagaaacgagaacgggagggaggactggcccctgcagtgcccccgggctccaacaaggaagctgcaggagatgcttcctccgaccagtccgcttgcacctccccagaagcttcacctaactcggtcaactcc

>HOXB2

Atgaattttgaatttgagagggagattgggtttataaacagccagccatcgcttgccgagtgcctgacttcctttcccgctgtcttggagacatttcaaacttcatcaatcaaggagtcgacattaattcctcctcctcctcctttcgagcaaaccatccccagactccagccgagcgcctccacccttcagagaccccggagtcaaaagcgaaccgaggatgggccggctccgccgcctccgccgccacagccgcctctcctggcagctcccctggcccccgaattcccctggatgaaagagaagaaatcgaccaagaaacccagccaatcggcctcatcccctgccccagcttcctccacagtgccggcctctggagctggatctccagatgcccctggacttcaggacggaggtggcagcggggcccggagactgcgcacagcctacacaaatactcagctgctggagctggagaaagaattccattttaacaaatacttgtgccggccccgccgggtggaaatcgcggcgctgctagacttgacagagaggcaggtcaaagtgtggttccagaaccggcggatgaaacacaaacgacagaaccagcataaggaacctctggatggggaagccgcttaccctggggctctggaggaggggagcgacccagcggaggagcccgaaggcagcccggccctgggtcaaactctggacgccccattgccatccttaggggataaagaggcctgttctctccccaccaagcccatcctaggtcagctcggacctcggttgctgtctgccccatcagagaattcgaatgctgagagtcctcagtaccccgaggacaggccaggggcgatagaaccggagactctcctggacagtgactcattccccgaagggcaagattctcccttcttgcccgatctcaacttctttgcttcagattcctgtcttcagctctcgggggggctttcccctagcctgcagggctccctggacagtccagtccgattctctgaggaagatctggatttcttcaccagcacgctccgaaccatagacttagaacacctacaattccac

>HOXB3

Atgcagaaagcgacctactacgataacacagcagctttgttcggaggttattcctaccctggcagcaatggcttcggttacgacgggcccacccagccccccttccaatccaccacgcatttggaaggagactaccagcgttcagcctgctcactccaagctctgggcaacacagtcccccatggcaaaagcaaagagttgaacggcagctgtatgaggcctggcttggccccggagcaccactcggccccccctccgggttctccccctcccagtgcagccccaaccagcaccagtagcaacagcaataatgccagtgggcccagcaaaaacgctccccctaaatgcggcccaggctccaactccaccctcagcaaacagatttttccctggatgaaagagtcaaggcaaaactccaaactgaaaaacagctcccccagcacagcagaaagctgcggcggcggcggtggcagtggtggaggcggcggaggcggagggggtgggggtggcggaggaggcgggggaggggacaagagcccgccgggatcctcggcctccaaacgagcccggacagcctacaccagtgctcagctggtggagctggagaaggagtttcatttcaatcgctatctgtgccggcctcgccgggtggaaatggccaacttactcaacctgagcgagcgccagatcaagatttggttccagaaccggcggatgaagtataagaaagaccagaagtccaaggggctgggttcgtcgtccgggggcccatctccggccggaagcccccctcagcccatgcaatctacagcgggcttcatgaacgccttacatactatgacctccagctatgagaccccgtccccccctgccttcggcaaaccccaccagaatgcctatgctttgccgtcgaactaccaaccgccactcaagggctgtgcagccccgcagaagtacacgcccaaccccgcgcctcccgagtacgaaccccacgtcctccaaggcaacgggggagcctacggtacaccgagcatgcaaagcagcccagtgtacgtcggggggggaggctacgtggatccacttccgccccctgctggcccctccttgtatggcctcaaccacctgtcccaccacccgtcggggaacctggactacaacggggcgccccctatggctcccagccagcaccacggaccttgcgacccgcaccccacctacacagacctttcttctcatcatgcgcctcctcagggtagaatccaagaagctcccaaactgacacatctg

>HOXB4

Atggctatgagttcttttttgatcaactccaactatgtggatcccaagttccctccctgcgaggaatattcacagagcgattacctacccagcaaccactcgcccgggtactacggcagcggccagaggcgagagagcagcttccagcacgaggcagggtacgggcggcgggcggcttgcaccgtgcagacctatgcgtcctgtcaagagcccggccacccgccgccgacggtgctgtccccgagggctccggtccagccgcaagcaggggcccttctccctgagccgggccagcgctgcgaggcggtcagccccagccccccgccaccgccctgtggtcaaaaccctcttcaccccagcccatcccactcctcttgcaaagagcccgtagtctatccctggatgcggaaagttcacgtgagcaccgtaaatccgaattacaacggaggagagccaaagcgctcccggacagcttacacccgccagcaagtcttggagctggagaaggagtttcactataaccgctacctaacccggaggcggagggtggaaatcgctcactctctctgtctctctgagcgccagataaagatctggttccagaaccgccggatgaaatggaaaaaagaccacaaattacccaacacgaagatccgatcaagcacgtcggggtcgtctggaggggcccaaagtcgagccaatgcaacccccacctcacta

>HOXB5

Atgagctcttactttgtaaactcgttctcggggcgctatccaaatggcccggactatcagttgctaaattatggcagtagcagctctttgaacggttcttacagggattctgctaccatgcataccagctcttacggctacaattacaatggaatggatctcagcatcaaccgctcctcagcctcctccagccactttggggcggtgggggagacctcgcgtgccttcccctcgcctggccaggagtcccggttcaggcaggcttcaagctgctccctgtcctctcctgagtccctgccctgctccaacagcgacaaccacggaggcaagccctctgcctcgtccccctccgaccaggcgacctctgccagttccaacaccaattttacagaaatagacgagaccagcgcgtcctcggagcccgaagaagcaagtcagttaaataacagcagtttaagccgagcccagacagaacccatcgcgacctccacagcgacaacggagggacagactccacaaatattcccctggatgaggaagctgcacattagccacgatatgaccggcccggatgggaaaagggcacggaccgcttacactcgctaccagaccctggagttggagaaagagtttcacttcaataggtatctcacccgccggcggaggatagagatcgcccacgctctctgcctctcggagcggcagattaagatctggtttcagaatcgccggatgaaatggaagaaagataacaaactgaagagcatgagtttggctaccgccggcagcgccttccagccc

>HOXB6

Atgagttcctattttgtgaactccaccttcccagttactctgtccaacggacaggagtcgttcctgggtcagctcccgctgtactcctcgggctatgcggatcctttaagacactatcccgcggcgtatgggaccggggcggtccaggacaagggttttgcctcttcttcctactacccaccggcgagcgggggctacaaccgggctgcggcttgtgactatgggccagccggcttctatagggagaaggagtctgcctgcgccctctccagcggcgaagagcctgcccccttccaccccgagccgaggaagtccgactgcgcccaagacaaaaatgttttcggagagagtgaggagcagaagtgttccactcctgtctatccctggatgcagcggatgaattcatgcaacagttcttcttttgggcccagcgggcggcgaggccgtcagacatacacgcgctaccagactttggagctggagaaggagttccattacaatcgctacctgactcgaagaagacgcatcgagatcgcccacgccctttgcttgaccgagagacagatcaaaatctggttccagaaccgacgaatgaagtggaaaaaggaaaacaagatgctcaactcttcccagctcagcccggaggcagaggaagaaaagcccacagag

>HOXB7

Atgagttcattgtattatgcgaatgctttattttctaaatatccagccgcaagttcggttttccctcccggagtcttccccgaacaaacttcttgcgctttcgcacccaacgcccagcgcccgggctatggagcgggctcgggcgcctccttcgctgcctccatgccgggcttgtaccccaacgggggggggatggcagggcagagcgcggccggcgtctacacggccggttacgggctggaggcgggttccttcaacatgcactgcgcgccctttgagcaaaatctctccgtgatgtgtcccggcgactctgccaaggcaggcggcggcaaggaccagagggagtccgacttggccgcggaaagtaatttccggatctacccgtggatgcggagcacagggacagaccgtaaacgaggccgacagacatatacccgctatcagactctggagctggagaaggagttccactacaatcgttacctaacccggaggagacgcatagagatcgcgcacgccctgtgcctgaccgagagacagatcaaaatctggttccagaaccgacggatgaaatggaaaaaggagaacaagaccacctgccccagctccaacaatcaggacaaaccagaggctgaggaagatgaggaggag

>HOXB8

Atgagctcttatttcgtcaactcactgttctccaaatacaaaactggggagtccctgcgccccaactattatgactgcgggttcgcccaggatctgggcggaagacccaccgtggtgtatggacccagtaccggaggcagcttccagcatccgacgcaaattcaggaattctatcacggggcgtcctcgctctccacgtccccgtaccagcagaacccgtgcgcagtggcttgtcatggggatccgagcaacttctacggttatgacccgctgcagagacagacgctcttcggggcccaggactcggacctggtgcagtatgccgactgcaagctcgccgccgccaacggcctgggggaggaagctgagggctcggagcagagtccttctcccacgcagctcttcccctggatgcgaccgcaagcagccgccggacgcaggcgaggcagacaaacctacagtcgctaccagacactggagctggagaaggagtttctgtttaatccctatctgactcgcaagcggcggatcgaggtgtcgcatgccctgggactgacagagagacaagtcaaaatctggttccagaaccggagaatgaagtggaaaaaggagaacaacaaagataagtttcccagcagcaagtgcgagcaggaagagttggaaaaacagaaaatggaacgggcccaggaggcggaggaagagggggaaggacagaaggcagacaaaaaa

>HOXB9

Atgtccatttctgggacgcttagcagttattatgttgactcgatcataagtcacgagagtgaagacgcgcctccagccaagtttccttccggccaatacacgagtcctcgccaagccggccacgcggagcatctcgaattcccctcctgtagtttccagcccaaagctccggtcttcagcgcctcgtggacgcctctgagccctcactcgtcggggagcctcccttccgtctaccacccgtatatccaacaccagagcgtcccgccttcggagagcaggtatctccgcacctggttggagccggtgcccaggagtgagggggggcccgggcagcccctagtgaaagccgaacctctgctggggggctcaggggagctccttaagcagggcaccccggagtacaatttagaaacttcttccggaagggaagctatcctatctaatcaaagacccggctacggagacaataaactttgcgaaggaagcgaagacaaagagaggcctgatcaaaacccctctgccaactggttgcatgcccgctcctccaggaaaaagcgatgcccttacaccaaataccagacgctggaattagagaaggagtttctattcaatatgtacctcaccagggaccgtagacacgaggtggccagactcctcaacttgagtgagagacaagtcaaaatctggtttcagaaccggcggatgaaaatgaagaaaatgaataaggaacaaggcaaagaa

>HOXB13

Atggagtccggtgcttatgccaccttggatgctggcaaagatatcgaaggcttgttgggagcgggcgggtgtcggaatctggtctcccactcgccactggcgagtcacccttcagcgcctgcgctactgcccacccccaactatacccccatagacctgcctggctccggggagccgccaaagcagtgtcacccatgccccggggtgccgcagggaggctcccccacatctgttccttatggctactttggtggaggctactattcctgtcgagtgtcccggagttcgctgaaaccatgcacccaggcagctaccctggctgcctatcccccggagaccactgcagccggggaggattatcctagccgtcctgcagaatttgccttttatcctgggtacccaggaccctatcagccggtggctagttacctggacgtgtctgtggtgcaaactcttggaggtcctggggagccccgccacgacaccctgttgccagtggacagttaccagccctgggccctcgccagcggttggagtggccagatgtgttgccaaggggaacagaaccaacccggtcatttctggaaggctgcgtttgcagtagacgcctcggcccagcatccacccgatggctgttcctaccgccggggccgcaagaagcggatcccgtacagcaagggtcagctgcgagagctggagcgggagtacgccgccaacaaattcgtcaccaaggacaagaggcgcaagatctcggcggccaccagcctttcggagagacagatcaccatttggttccagaaccgccgggtcaaggagaaaaaggtcttagctaagatcaagaccaacgccaccccc

>HOXC4

Atgatcatgagctcctatttgatggactctaactacatcgatccgaaatttcctccatgcgaggaatattcgcaaaatagctacatccctgagcatagtccggaatattacggccggaccagggaatcaggatttcagcatcaccatcaagagctgtacccaccaccacctccacggtctagctatcccgagcgccaatatagctgtaccagtctccagggaccgggcaatccaagaggccacgggccggctcaggcggggcaccatcaccccgagaaatcgcagccgctctgcgagccagctcctctctccggctcctccacttccccctccccagccccgccagcctgcagccagccagccccagaccatccctccagcgccgcatccaagcagcccatagtgtatccatggatgaaaaaaatccacgttagcacggtgaaccccaattataatggaggggaacccaagcgttcgaggacagcctacacccgccagcaagtcctggaattagaaaaagagtttcattacaaccgctacctgacccggaggagaagaatcgagatcgcccactcgctgtgtctctccgagaggcagatcaaaatttggttccaaaaccgtcggatgaaatggaagaaggaccaccgactccccaacaccaaagtcagatcggccccaccagccggagcggcgcccagcgccctgcccgccaccacccccggccctactgaagaccattcccaaagctcaacaccacaggagcagcgagcagaggacattaccaggtta

>HOXC5

Atgagctcctacgtagccaattcattctataagcaaagccctaatattccggcctatagcatgcaaacttgtgggaactatggatctgtctcagaggtgcagccatccaggtactgctacagtggattggacttaagcatcactttcccaccgcctgctccttctaactctctccacgggttagacatggctgccactcctagacctcacccagaccgacccgcctgtaccacggtggcagctccgggacacgctctgggcagagacgaaccggctcctctaaaccccggaatgtacaatcagaaggcggctcgcccggcgctggaggacagatctaaggggagtggggagatcaaggaagaacaggcgcagactgggcagcccgcaggactgagccagccaccggccccgccacaaatatacccgtggatgaccaaactccacatgagccacgagacagacggcaagcgatccagaaccagttacacgcgctaccagactttggaattagagaaagaatttcactttaaccgctacctcacccgccgaaggcgaattgagatcgctaacaacttgtgtctcaacgagagacagatcaagatctggttccagaaccgaaggatgaaatggaagaaggattccaaattaaaaagcaaagaggctctt

>HOXC6

Atgaattcctatttcactaacccgtccttatcgtgccatctcgccgggggtcaagacgttctccccaacgtcgccctcaattccaccgcctatgatccagtgagacatttctcgacctatggagcggcggtggcacagaaccggatctactcgtctcccttttattcgccacaagagaatgtcgtgttcagttcgagccgggggccgtatgactatggatcaaattccttttaccaggaaaaagacatgctctcaaattgcaggcagaacaccttagcacacaacacacagacctcaatggcccaggactttagctctgagcaaagcaggactgctccccaagaccagaaaaccagtatccagatctacccctggatgcagcgaatgaattcgcacagtggggtcggctatggggcggacaggagacgcggccggcagatctattctcgataccagaccttggagctggaaaaagaatttcacttcaaccgttacctaacccggcgccggcggatcgagatcgccaatgcgctctgcctgaccgagagacagatcaaaatttggttccagaaccgccggatgaaatggaaaaaggaatctaatctcacttccacgctgtcggggggcggcggaggggccgcggccgacagcctaggcggcaaggaggagaagcgggaagagacagaagaagaaaaacagaaagag

>HOXC8

Atgagctcctactttgtcaaccccctgttctccaaatacaaaggcggtgagtccttggagccggcatattacgactgccggttccctcaaagtgtgagtaggagccatgccttagtatacggcccgggcagcacggcgcccagctttcagcacgcctcgcaccacgtccaagacttcttccaccacgggacttcgagcatctccaactccggctaccagcagaacccgtgctccctcagctgccacggagacgcctccaaattctatggctacgaggcgctccccagacagcctctttatggggctcagcaagaggcgagcgtcgtgcaatatcccgactgtaaatcctccgccaacagtaacagtaacgaaggacaagggcacctaaatcaaaactcgtctcccagtctcatgtttccatggatgagaccccacgctcctgggcggcgcagcggacgacaaacttacagccggtatcagaccttggaactagaaaaggagtttctctttaatccatatttgacacgaaagcgacggattgaggtctctcacgccctgggactgactgaaagacaagtgaagatttggttccagaaccggaggatgaagtggaaaaaggagaataacaaggataaacttcctggagcccgagatgaggagaaaacagaagaagaggggaacgaggaagaggagaaagaagaggaagaaaaagaagagaacaaggac

>HOXC9

Atgtcggcgacgggacccatcagtaactattacgtggactccctgatcagccacgagaatgaagatctcctagcgtccaggttccctgccactggggctcaccccgcaaccgccagacccaccggcttggtcccggactgtagcgattttccgtcctgtagttttgcccccaagccggcagtgttcaccacgtcctgggctccggtccactcccagtcgtctgtcgtctaccacccctacacccaccagccccaccttggagcggacacgcggtatatgcggacttggctggagcccctatccggggccgtctccttccccagctttccagctgggggccgccactacgccctcaaacccgacgcctacccgggccgccgggcggactgtggcccgggggacgggcggagttacccagactacatgtatggctctcccggggagcttcgggaccggaccccgcaggctctcccctccccggagtcggacgcgctggccagcagcaagcacaaagaggagaagacagaactggacccgagtaacccagtggcaaattggatccacgctcgctctacgaggaagaagagatgtccctacaccaaataccagacactggaactggagaaggagtttctgttcaatatgtatttaaccagggaccgtcggtacgaggtggcccgggtcctcaacctcaccgagcgccaggtcaaaatctggtttcaaaaccggaggatgaagatgaaaaagatgaacaaggagaaaaccgacaaggaacaatcc

>HOXC10

Atgacatgccctcgccatgtaactcctaactcgtatttggagcccctggctgcgtcttgcagagcggagacctatacgtccaacccagggatgtacatgcaatccgggagtgacttcaactgtggggtgatgaggaactgtgggatcgtgccctctctctccaagagagacgagggcggcagccccagtctctctctcaacacctacccgtcttacctttcgcagctggactcgtggtgcgatcccaaaaccgcgtaccgtatagaacaacctgttggcagacagctgtcgtcctgctcctacccaactagtgtcaaggaggagaatgtgtgttgcatgtatagcgccgagaaaagggcaaaaagtggtcctgagacagccctctaccctaaccccttgtccgagtcctgcctcggggagcatgaagtccccgtccccagctactatagagccagccagagctaccccaccctggagaaaccgccccactgttccggggcgaccgagttcgaggcgagtttcgaaccccgggctagcctccatccccggagtgaacatctggaatcgcctccgcttggggggaaagtgagtttcccggagactcccaagtcggacagccagaccccaagccccaacgaaatcaaaacggagaagagcctggtaggacccaaaaccagcccttcggaaagcgagaaggagctgagtaagactacagacaccagtaccgataattcggataacgaagcgaaagaggatataaaggcagaaaacacgacaggaaattggctgacagcaaagagcggaaggaagaagaggtgcccctatactaaacaccagacgttggaactggagaaagagtttctgttcaatatgtatttgacccgagagcgccgcctggagattagcaagacgattaaccttacagacagacaagtcaaaatctggtttcaaaatcgcagaatgaaactcaagaaaatgaaccgagagaatcgaattcgcgaactgacctccaattttaatttcacc

>HOXC11

Atgtttaactcggttaacctgggtaacttctgctcgccgtcgcgcaaagagaggggcgctgatttcggcgaaagagggagctgcgcctccaatctctatctgcccagctgcacttactacgtgcccgagttctctaccgtctcttctttcctgccccaggccccctctcgccagatctcctacccttactccacccaagtgcccccagtccgggaggtctcctatggcctggagacctcggggaaatggcatcacagaaacagctactcttcctgctacgctgcggacgagctcatgcacagggaatgtctccctccttccaccgtgaccgaaatcctcatgaaaaacgaaggctcctacggcagccatcaccaccccagcgccccccacccgggtcccggcttctattcgtcagtcaacaagaacagcgtcttgccccaagccttcgaccgattcttcgacaatgcctactgtgggagcgacgggccggccgagcccccttgcgtgggcaagggagaaggcaagggggaacccgatcccccccagaccggggtcctagctccccggcccgagtccggcggagacccggaggacgaggaggagaacactaaccccagctcatccggctcgtcccactcagccagcaaagaggctagcaagggaaccaacgccccccgcacccgcaagaagcgctgcccttattcgaaattccagatccgggaactggagagagaatttttcttcaacgtctatatcaacaaagagaagcggctgcagctgtcccggatgctgaacctgacagaccgacaagtgaagatttggtttcagaacagaagaatgaaagagaaaaaactgagcagagaccggctgcagtatttctctgggaaccctctactg

>HOXC12

Atgggcgaacataatctccttaatcccgggtttgtggggccgctggtgaatatccacacgggggacaccttctacttccccaacttccgcgcctcgggggggcagctgcccggcctgccttccctgtcctaccctcggcgtgacaacgtctgctccctgccttggccgtcggcggagccgtgcaatggctatccccagccctacctcggcagccccgtgtcgcttaacccgtcctttggccggacgtgcgagctggcccgggtggaggagagcaaatgctactatcgcgaggcgtgcgccgagggcggtgggctgaagcgcgaggagcgggggcgggaggggggcgggttgctgcagctcgagccgtcggggccgccgtctatgggattcaagtacgactacgctgcaggcggcggcggcggtggcggcgggggggacggagccgcggggcccccccacgatcccccctcctgtcagtcactcgagtccgattccagttcgtctctgctcaacgaaggaaacaagggtggcggcgcgggcgatggggccagcctggtgtcgcctctaggccaggggaacgggctctccaccagtggcgccccttggtatcccatgcacacccggtcccggaagaagcgcaaaccctactcaaagctgcagctcgcggagctggagggagagttcatggtgaacgagttcatcacccgccagaggaggagagaactctcggaccgcttgaatcttagtgaccagcaggtcaagatctggttccaaaaccggaggatgaaaaagaaaagacttctgcttagggagcaggccctctccttcttt

>HOXC13

Atgacgacttcgctgctcctgcacccgcgctggccggagagccttatgtacgtctatgaggacagttcggcggagagcagcggaggaggaggcggcgccggcggcggaggaggcgagggaggcggaggcggcagctgcggaggaggcggcggaggggggggaagctgcagcggagccagccccggcaaagcccctatcatggacggactgggcggcagctgcccctccagccactgccgggacctgctcacccaccaggttctgggccggcccccggctcccctgggcgccccacagggcgccgtctacacggacatccccgccccagaggcggcccgccaatgccccccgccgccagcgcccccaacctcgtccagcgctaccctcggctacggctacccgttcgggggtagctactacggctgccgcctgtcccacaacgtgaacttgcagcagaagccctgcgcctaccaccctggggacaaatatcccgagccgtcgggggccttgccgggagacgatttatcctccagggccaaggagttcgccttttatcccagctttgccagctcctaccaagcgatgcccggctacttggacgtgtccgtggtaccgggcatcagcgggcaccccgagccccgccacgacgccctcatccccgtcgagggttaccagcattgggcactctccaacggctgggacagtcaggtgtattgctccaaggagcagtcgcagtcagcccacctctggaagtctccttttccagacgtagtccccctgcagcctgaggtcagcagctaccgaaggggacgaaagaagcgagtgccctacaccaaggttcagctgaaggaactcgagaaggagtatgcggccagcaagttcattaccaaagagaagaggcggcggatctcagccactaccaacctctcagaacgccaggtcaccatctggttccaaaaccgtcgagtcaaagagaaaaaggtggtcagcaaggctaaggcaccccatctccactctacc

>HOXD1

Atgaattcgtacctggagtacatctcgtgcggagaggtgctcaccttctcccccaagttctgccgctcggatccccggccagcggctctgcagcctgcctttcccctgggcggggagggcgcctttgtcagctgcctgcccgtcgccgcggctggggctcggccggtgccttctgccccggcgcagcctcaaccccagcctccgacgccaacgccgcccccgccgcagctgccacagcccccagccccgtccagggcccccggcccgcctacgtttgctcagtgcaccctcgaaggcgcctacgaggccggcgccgcacctgccgcacactacagctttctgccacagggctccgcatacgactttgccggcggcctagggcccacgaccgaagacggcgtggccggccaagttcactacgccacttcggccgtcttttcgggcggcggctctttcattctcagcggccaagtggactacggcgccttcgcagagcccagcgccttcccacagggtcttaaggagcaaggcgacggtcccgccgggggcttccaggctgtctccccagcgccgggctcctaccctaagtccgtctctccggcctcggggcttcccacagccctcagcacttttgaatggatgaaagtgaaaagaaacgcccccaagaaaagcaaactctccgagtatggagtacacagctcctcaagcacaatacgcacaaatttcagcaccaagcaacttactgaactggaaaaagagtttcatttcaataagtacttaacccgggcccgtcgcatagaaatagcccactctctgcagctgaatgacactcaagtcaaaatctggttccagaaccgaagaatgaaacagaagaaaagggaaagagagggattattggccaacacatcacctgtaacatctcttcagctttctgtctcaggtatgagccctgcaaagtctggcaagaatgcagagagccctgctccctccaaagactcttct

>HOXD3

Atgttatttgagccgggtcatcaggccccagaaatcccagagtgcacgatgcagaagactgcctactatgagaacacaggcctctttgggggttatggctacagcaaggctactgatacttatggttatgggtccactcatcagccttatccccctgctgcccctgcagccctggaagctgactatacaggttctgcttgctccatccagagctcagccccaatccgtgccccagtccacaaaagtggggagctcaatggtagctgtatgcgtccaggaggtgggagtgggcagactggtgggggtggtagtcaaccccctggcttgaacacagatcagcagccaccaccaccacctcccccaccacccacactgcccccatcctcacccacaaatcctggtagtgctaccccagccaagaaggccaagggtggccccaatgcctcaggctcttcctctgctactatcagcaaacagatcttcccctggatgaaggaatcccggcagaactccaagcagaagagcaattgtaccacttcaggtgaaagctgtgaggacaagagccctcccggcccggcatccaaacgggttcgtacagcttacacaagtgcgcagctggtggagttggagaaggaatttcatttcaaccgctacttgtgccgcccacgccgggtggagatggccaacctgctcaacctcaccgagcgccagatcaagatctggttccagaaccgacggatgaaatacaagaaggaccagaaggcaaagggcatcatgcactcgcctgtgggtcagtctcccgagcgcagccctccactgagtggggctaaccacgtgggctactcaggccagctccccccagtgccggggctgggctacgacgctccctcacccccatctttcgccaagtcccagcagaacatgtatggcctggccgcctacacagcgccgctcagcagctgcctaccacaacagaagcgatacccaggacaggagtacgaccaccaccccatgcagagcaacgggagttttgccaacgccaacctgcagggcagcccggtctacgtcggtgggaactttgttgattccatgccggcctcggggccagtcttcaacctgggccacctctcccacccctcgtcggccagcgtggactacagctgcgccgcccagatcccgggcaaccaccatcacggaccgtgtgacccccaccccacctatacagatctcacgtctcaccatacgtctcagggaaggattcaagaagcccccaaactgacgcatctg

>HOXD4

Atggccatgagttcgtatatggtgaactctaagtatgtggaccccaaatttcctccttgtgaggaatatttgcagaatagctacctagccgagcagagctcagactattatagtggctctcagggttcggatttccagcatcagggagtctaccctcggtcaaactacagcgagcagcccttcagctgtagcaatgcccaaggctctacggtgcctccgcggggtcatggacaggagccatcgggcccggcaagccactaccctggccaaggggagcattgtcccccgcctccaattcccagttcccgggcctgcagccagtcggccagcctcaagcaacctcccaacgggacggccctcaaacaacccgctgtggtgtacccctggatgaagaaagtgcatgtaaattctgtgaaccctaattacaccggaggggagcccaagcggtccagaacagcctacacaaggcagcaagtcctagaactggaaaaggaattccattttaacaggtatctgacgaggcgtcgtcggatcgaaatagcccacactctctgtctttctgaacgccagatcaagatctggtttcagaaccgtaggatgaaatggaaaaaagaccacaaactgcccaacacgaagggcaggtcttcctccgccgcctcgaatcagcatttacagccggtgtccaaggaccatcacactgacttgacgacttta

>HOXD8

Atgagctcctactttgtgaacccgctgtactccaagtacaaggcggcggccgcggcggcggcggcggcggcggcggcggcggcgggcgaagccatcaatcccacttactacgactgtcactttgcacccgatgtcagcggccgccacgccgccgccgccgccgccgccctgcagctgtatagtaacagcgccgccggcttccagcaccaccaccaccaccaccaccaccaccagccgcagcagccgccccagcagcagccgcagcagcagcctcacccgggatccgctagcggagggggcggcggcggcgggggcccggactattaccatcccgccggggggagtccggcctctgcctaccagccggcccccccccctccacatcctcctcctccgcctcctcctccgccgccgcctcccccctgcagtgggattgcctgtcacggggagcccgctaagttttacggatacgataacttacagagacagccgatttttacgacccagcaagaggccgagctggtacaatatcctgactgtaaatcgtccagtggtaatattggcgaggacccagaccacttaaatcagagctcgtctccttctcaaatgtttccctggatgagaccacaagcagctcctggtaggaggagaggaagacaaacgtacagtcgtttccaaacgctagagctggaaaaggaattcctttttaacccttatctgaccaggaagagaaggattgaggtttcccacgctctaggactcaccgagaggcaggtaaagatatggtttcagaacaggagaatgaaatggaaaaaagagaacaacaaggacaaatttccagtttccagacaggaggggaaggaaggggaaacaaaaaaagaagcacacgatctggaagaggatagagctgagggccagacaaat

>HOXD9

Atgtcgtccagtggcaccctaagtaactactatgtggactctctcataggccacgagagcgatgaagtgtactccgggcgcttcgggcaggcggggcacggcacggccgccaggccgtcaggtgtggccgatgggaccgaattctcctcctgtagctttgcccccaaatccaccgtgttctccgcctcctggcccaccgtgcccccacagccctcggcggctatgacagggatctaccatccctacgtgcatcagccccatttagcagcctccgagcccagtcggtacgtgcgctcctggatcgagcccttcccgagtttccccagcagcagcacgggcagcagcggcaccgggagcagcggcggcggcggcggcggcggcacgtcctccaccgggcgccactacggaattaagcctgaaacaggagtagctcctgctacctcctcctcctcctcatcctcctcttcttcttcctcttcctcctcctgtccctcctcctccaccacctcctcctcctcctcttctaccaaaaggactgagtgctcctcgtcccgggagtcccaggggatcagtgtccccgagtacacgtgcaattccttcctgcaggagccccgggagaaggcagcagcggcagcgacagcagcggcagttgccggagtggccaacagcaaggagccctcggcttgcagcgaccaccccagcccgagcagccagctgaaggaggagaagcagcagcaacagcagcagcaaccacaacagcagcaacttgacccaaaccctgcagcaaactggatccacgcacgttccaccaggaaaaagcgctgtccctacacaaaataccagaccctggagctggaaaaggaatttctctttaacatgtatctcactcgagaccgccgctacgaagtagctaggattctaaacctcacagaaaggcaggtcaaaatctggtttcaaaaccgaagaatgaaaatgaaaaagatgaacaaggaaaagggcagtaaaggagac

>HOXD10

Atgtcctttcccaacagctctcctgctgctaatacttttttagtagattccttgatcagtgcctgcaggagtgacagtttttattctagcagcgccagcatgtacatgccaccacccagcacagacatggggacttatggaatgcaaacctgtggactgctcccgtctctggctaaaagagaagttaatcaccaaaatatgggtatgaatgtgcatccttatatacctcaagtagagagttggacagatccgagcagatcttgtcgaatagagcaacctgttacacagcaagtccctacttgctccttcactgccaatattaaggaagaaaccaattgctgcatgtattccgataagcgaaccaaactaatttcttccgacgtcccttcttaccagaggttggtctctgaatcatgccccattgaaaatcccgaggttcctgtcccaggatattttagactgagccagacctacgccactgggaaaacccaagagtacaataacagccccgaaacgagttcaactgtaatgttacagttaaaccctcgcggctctgccaaaccgcagctatcttctgcccaccttcagatggaaaagaaaatgagtgaaaaccccagcaaccaagactcagctaaagtccctcaagtggaaagccccgagcccaagtccactttgccagaggagaggagctgcctagctgaagtctccgtgtccagcccagaagtccaagagaaggaaagtaaagaggaaatcaagtccgatactccaaccagcaattggttaactgcaaagagtggcagaaagaagaggtgtccttatactaaacatcaaacactggaattagaaaaagagttcttattcaatatgtacctcactcgcgagcgccgcctagagatcagtaagagcgttaacctcactgacaggcaggtcaagatctggtttcaaaaccgcagaatgaagcttaagaagatgagcagggagaaccgaatccgagaactgaccgctaatctgaccttctct

>HOXD11

Atgaccgagtttgacgactgcggccccagcgcagccaatatgtatctgccgggctgcgcttattacgtggccccctcggatttctccagcaagccctcgttcctgtcgcagacgtcgtcttgtcaaatgactttcccctactcctccaacctgcctcacgtccaaccggtgcgcgaagtggcgttccgcgactacggcttggagcgcggtaaatggcaataccggggcagttacgctccctattacgcggcggaggaggtgatgcacagggacttgctgccgcccgggagccggaggccggatatgctattcaaagctgccgaacccgtttgcggtccccacggcccctcgggcgccgcctccaacttctacagcaccgtggggcgcaatgggattctgccccagggcttcgaccagttttacgaggcggcgcctggaccagcgtaccagcagcagcagcagcaacaacctcagcagcctcagcagcaacagcaacagcagccgcctcagcccagcggcgagcccgagggggacgctgagaagagcggtctgaagtctccggccactgcgagtccctgcggcaaagccccctcggggccggagcccaagggggcagcggagagtggctccagtggcgaggcccccccaggagaggcgggggcggagaagagcggaggcgcagcagtggttccccagcgatcgaggaaaaagaggtgtccctacaccaaataccagatccgagaactggaacgggaatttttcttcaacgtgtatataaacaaagagaaaagactccaactgtctcgaatgctcaacctcactgaccgacaagtcaaaatctggttccagaatcgcaggatgaaagaaaagaaactgaacagagatcgactccaatatttcactgggaaccccttattc

>HOXD12

Atgtgcgatcgcagtctctacagatccgggtatgtggggtctcttctgaatttgcaatcaccagattctttctatttctccaaccttcgggcgaatggcagccagttggcggctctgccccccatctcctacccccgaggctctctgccttggtcagcggccagctcctgcgccgctcaacccccaagccactctgccttcggcggctatacccagccttacctcccaggctccgtgcccctcaacctccaccccccgggcagcaaggagggcccagaagatcaaggcaaatactatggttccgagtctggttcgaggccagaggagcgtagccgtcccaggccggctttcaccaacgagcctagcctggctccaggcgttggcctcaagccggccaagtacgactactcaggcatggcgcggagcgtgcacgggtcagcggctcttttggaagggaacccctgtgccgcaggcttcaaggacgacgccaagggctcgctcaacttgaacgtgacagtgcaggcggccaacttagcctcctgccttcgaccttcgctacccgacggattgccctggggggcggcccaggggagagcccgcaagaaacggaagccctacactaagcagcaaatcgcagaattggaaaacgaattccttctcaacgagttcatcaatcgtcagaagcggaaggagttgtccaacagactcaatctgagcgatcaacaggtcaaaatctggttccagaaccggcggatgaagaagaaaagagtggtgctgagagaacaggcactcactctctac

>HOXD13

Atgagccgggctggaagctgggacatggacgggcttcgagcggacggcggcgcgaccagcgggggcgcccctcctcgcggccgctactgccgggcagtggcggcggcttccttccttcgaccagtattcggagcacactctggccgggcagccgcagcggcggcggctgcggccgcggcagctgcagcggcttcgggcttctcctatcccggggctgcagaacgctccggttcatcctcttcctcctcgtcgtccgcagtggcagcggcggctgctgcagcgcgtcccgaagcaccccctaccaaggattgtcccggctccactccagctccgactccagctgcagcgcccccggccgccccggcattgggctacggttatcattttggtaatggatactacagctgccgcatgtcccacggcgtggggatccaacagaacgcactcaagtcctccccccacgcctctctggggggctttcccgtggaaaaatacatggacgtctccagcctggccagcaccagtgtcccggccaacgaggtcccctcccgagctaaggaagtgtccttttaccagggctacaccaacccttaccagcacgtccccgggtatatagacatggtctccacctttggctcaggggaaccgagacacgaaacgtacatatccatggagggttaccagtcctggactctggcgaatgggtggaacagtcaggtttactgtgctaaagaccagtcccagagctcacatttttggaaatcttcctttccaggggatgtagctctaaaccagcctgatatgtgtgtctacagacgtggaaggaagaaacgcgttccatataccaaactgcagctgaaagaactggagaacgaatacgccattaacaagttcattaacaaggacaagaggagaaggatatccgcagccacaaacctgtctgagagacaagtgaccatttggtttcagaacagaagagtgaaggacaagaaaatagtctccaaactcaaagacaatgtctcc
