# Supplementary material for: Evolution of coding and non-coding genes in HOX clusters of a marsupial
Source: BMC Genomics. 2012 Jun 18;13:251. doi: 10.1186/1471-2164-13-251 (PMC3541083; doi:10.1186/1471-2164-13-251)
Supplement: Additional file 3 — Repetitive elements in tammarHOXclusters. [file 1471-2164-13-251-S3.doc]

**Additional file 3:** repetitive elements in tammar HOX clusters

|  | | Number of elements | | | | Length occupied (bp) | | | | Percentage of sequence (%) | | | |
| --- | --- | --- | --- | --- | --- | --- | --- | --- | --- | --- | --- | --- | --- |
|  |  | HOXA | HOXB | HOXC | HOXD | HOXA | HOXB | HOXC | HOXD | HOXA | HOXB | HOXC | HOXD |
| SINEs | | 3 | 71 | 13 | 32 | 198 | 9713 | 1727 | 5198 | 0.17 | 4.67 | 1.19 | 3.81 |
|  | Alu/B1 | 0 | 0 | 0 | 0 | 0 | 0 | 0 | 0 | 0.00 | 0.00 | 0.00 | 0.00 |
|  | MIRs | 0 | 45 | 8 | 25 | 0 | 5459 | 1256 | 4093 | 0.00 | 2.63 | 0.87 | 3.00 |
| LINEs | | 0 | 66 | 3 | 31 | 0 | 38304 | 257 | 10703 | 0.00 | 18.43 | 0.18 | 7.85 |
|  | LINE1 | 0 | 36 | 1 | 10 | 0 | 27923 | 67 | 3560 | 0.00 | 13.43 | 0.05 | 2.61 |
|  | LINE2 | 0 | 19 | 1 | 10 | 0 | 5127 | 112 | 1268 | 0.00 | 2.47 | 0.08 | 0.93 |
|  | L3/CR1 | 0 | 6 | 1 | 4 | 0 | 1372 | 78 | 929 | 0.00 | 0.66 | 0.05 | 0.68 |
|  | RTE | 0 | 5 | 0 | 7 | 0 | 3882 | 0 | 4946 | 0.00 | 1.87 | 0.00 | 3.63 |
| LTR elements | | 8 | 12 | 11 | 3 | 759 | 1492 | 680 | 194 | 0.64 | 0.72 | 0.47 | 0.14 |
|  | ERVL | 0 | 0 | 0 | 0 | 0 | 0 | 0 | 0 | 0.00 | 0.00 | 0.00 | 0.00 |
|  | ERVL-MaLRs | 1 | 0 | 0 | 0 | 70 | 0 | 0 | 0 | 0.06 | 0.00 | 0.00 | 0.00 |
|  | ERVL-class I | 4 | 5 | 4 | 1 | 436 | 351 | 159 | 75 | 0.37 | 0.17 | 0.11 | 0.06 |
|  | ERVL-class II | 3 | 7 | 7 | 2 | 253 | 1141 | 521 | 119 | 0.21 | 0.55 | 0.36 | 0.09 |
| DNA elements | | 0 | 12 | 2 | 3 | 0 | 1881 | 172 | 527 | 0.00 | 0.90 | 0.12 | 0.39 |
|  | hAT-Charlie | 0 | 9 | 2 | 2 | 0 | 1387 | 172 | 288 | 0.00 | 0.67 | 0.12 | 0.21 |
|  | TcMar-Tigger | 0 | 0 | 0 | 0 | 0 | 0 | 0 | 0 | 0.00 | 0.00 | 0.00 | 0.00 |
| unclassified | | 0 | 0 | 0 | 1 | 0 | 0 | 0 | 170 | 0.00 | 0.00 | 0.00 | 0.12 |
| Total interspersed repeats | | - | - | - | - | 957 | 51390 | 2836 | 16792 | 0.81 | 24.72 | 1.96 | 12.32 |
| Small RNA | | 2 | 1 | 1 | 0 | 155 | 269 | 67 | 0 | 0.13 | 0.13 | 0.05 | 0.00 |
| satellites | | 0 | 0 | 0 | 0 | 0 | 0 | 0 | 0 | 0.00 | 0.00 | 0.00 | 0.00 |
| Simple repeats | | 47 | 64 | 51 | 48 | 2697 | 3384 | 3327 | 2561 | 2.27 | 1.63 | 2.30 | 1.88 |
| Low complexity | | 53 | 54 | 46 | 24 | 3488 | 3323 | 2577 | 1183 | 2.94 | 1.60 | 1.78 | 0.87 |

Note: ERVL, endogenous retrovirus L; hAT, first letter of three “founder” transposons (hobo, AC and Tam3); L3/CR1, LINE3/complement component receptor 1; LINE long interspersed repeat element; LTR, long terminal repeats; MaLR, mammalian LTR; MIR, mammalian-wide interspersed repeats; RTE, retrotransposable element; SINE, short interspersed repeat element;
