# Supplementary figures and images for: Evolution of coding and non-coding genes in HOX clusters of a marsupial
Source: BMC Genomics. 2012 Jun 18;13:251. doi: 10.1186/1471-2164-13-251 (PMC3541083; doi:10.1186/1471-2164-13-251)

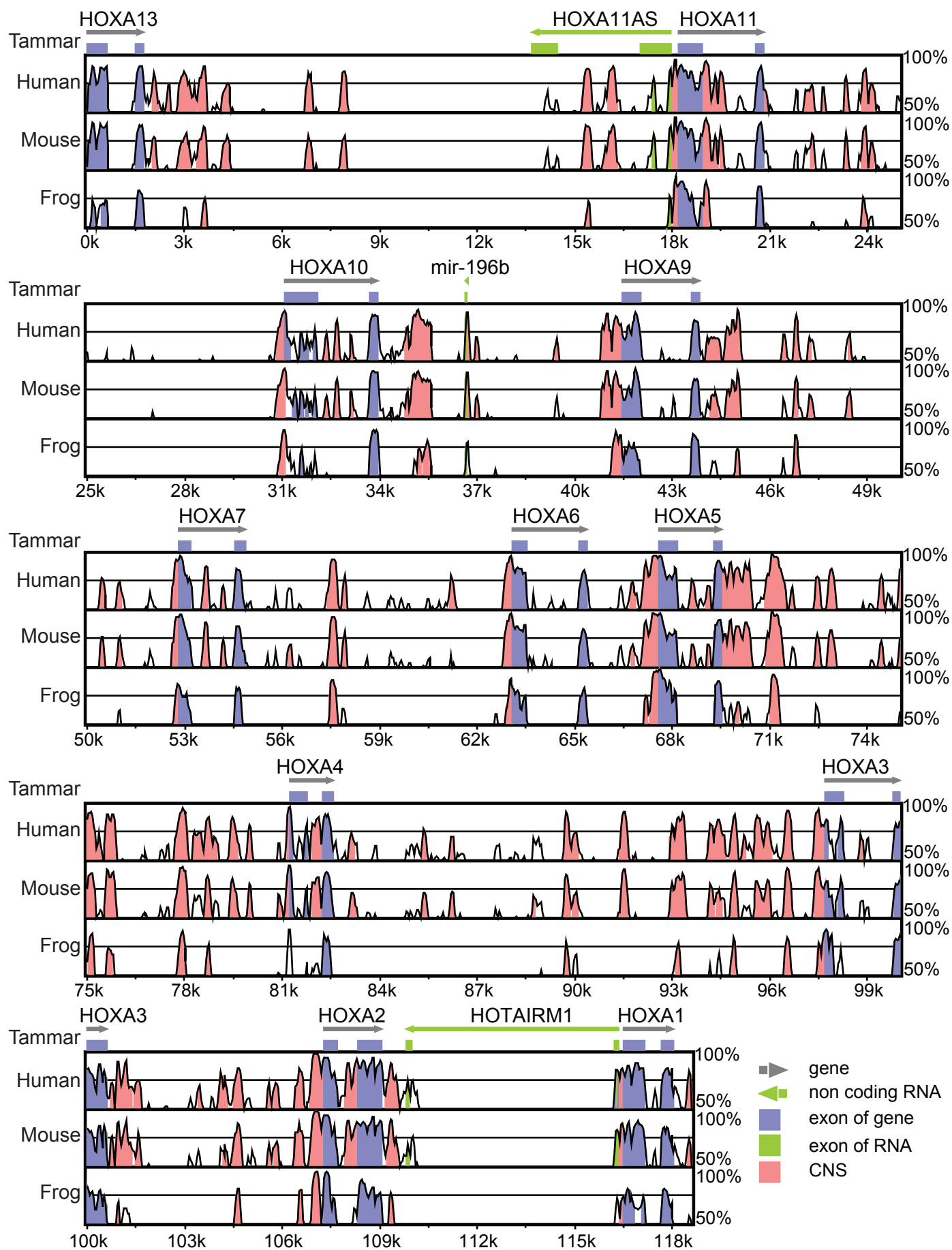

Supplement: Additional file 4 — Phylogenetic footprinting analyses ofHOXA cluster with mVISTA. mVISTA plot generated with HOXA genomic sequences from tammar, human (chr7:27131531–27244164), mouse (chr6:52104079–52216539) and frog (scaffold_56:1381000–1485000) with tammar as a reference. Conserved regions above the level of 70%/100 bp are highlighted under the curve, with red indicating conserved non-coding regions, blue representing conserved coding-protein exons, and turquoise representing microRNAs or long non-coding protein exons. HOTAIRM1 and HOXA11AS representing the long non-coding RNAs are conserved in all mammals and have much lower similarity in frog. microRNA miR-196b is highly conserved in all species. Arrow stands for the transcription orientation. [file 1471-2164-13-251-S4.pdf]

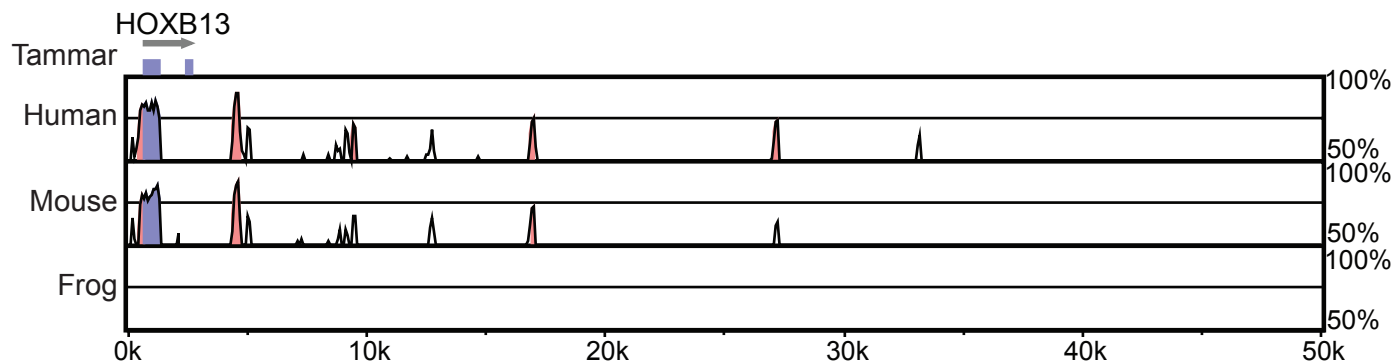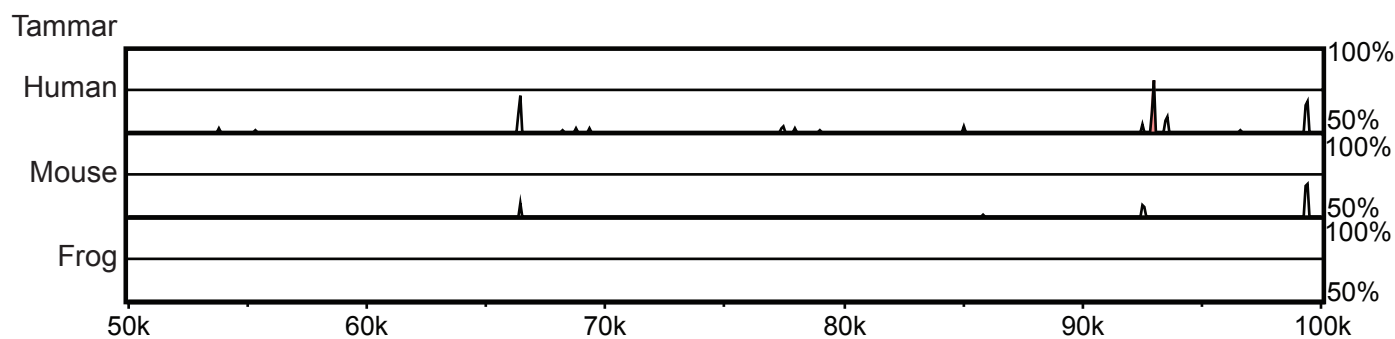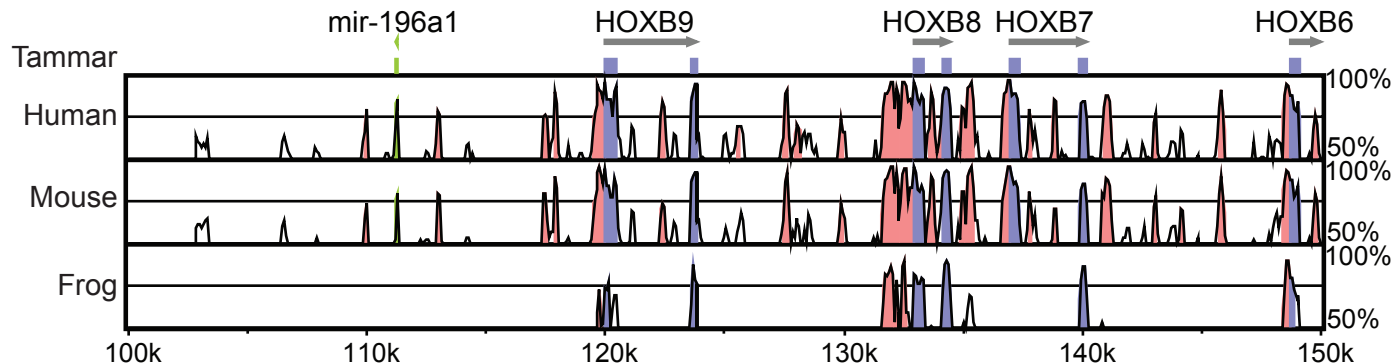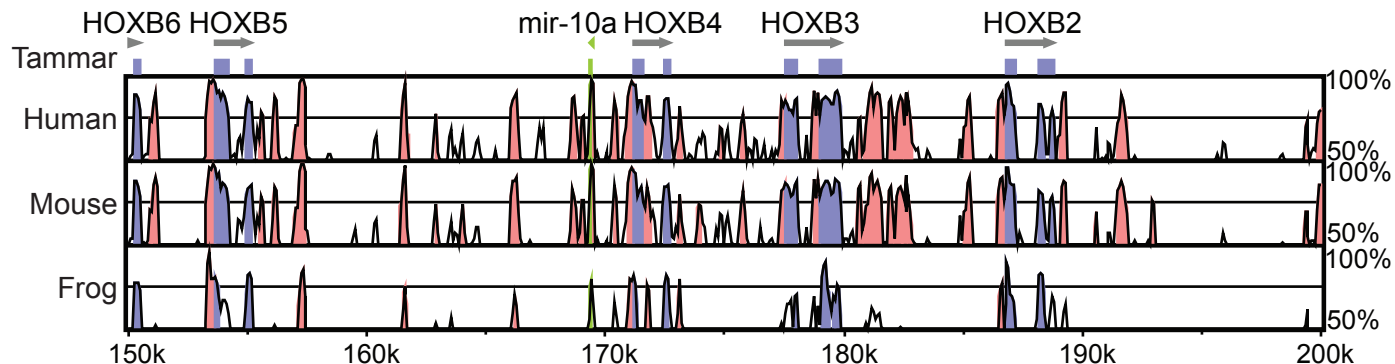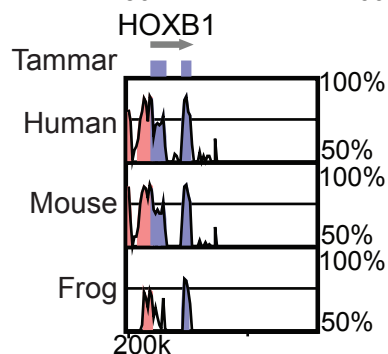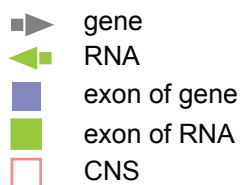

Supplement: Additional file 5 — Phylogenetic footprinting analyses ofHOXB cluster with mVISTA. mVISTA plot generated with HOXB genomic sequences from tammar, human (chr17: 43960868–44165742), mouse (chr11: 96024912–96229585) and frog (scaffold_334: 483000–620000) with tammar as a reference. microRNAs miR-10a located between HOXB4 and HOXB5 is highly conserved in all species. miR-196a1 is also conserved in all mammals. Other details as in figure Additional file 4. [file 1471-2164-13-251-S5.pdf]

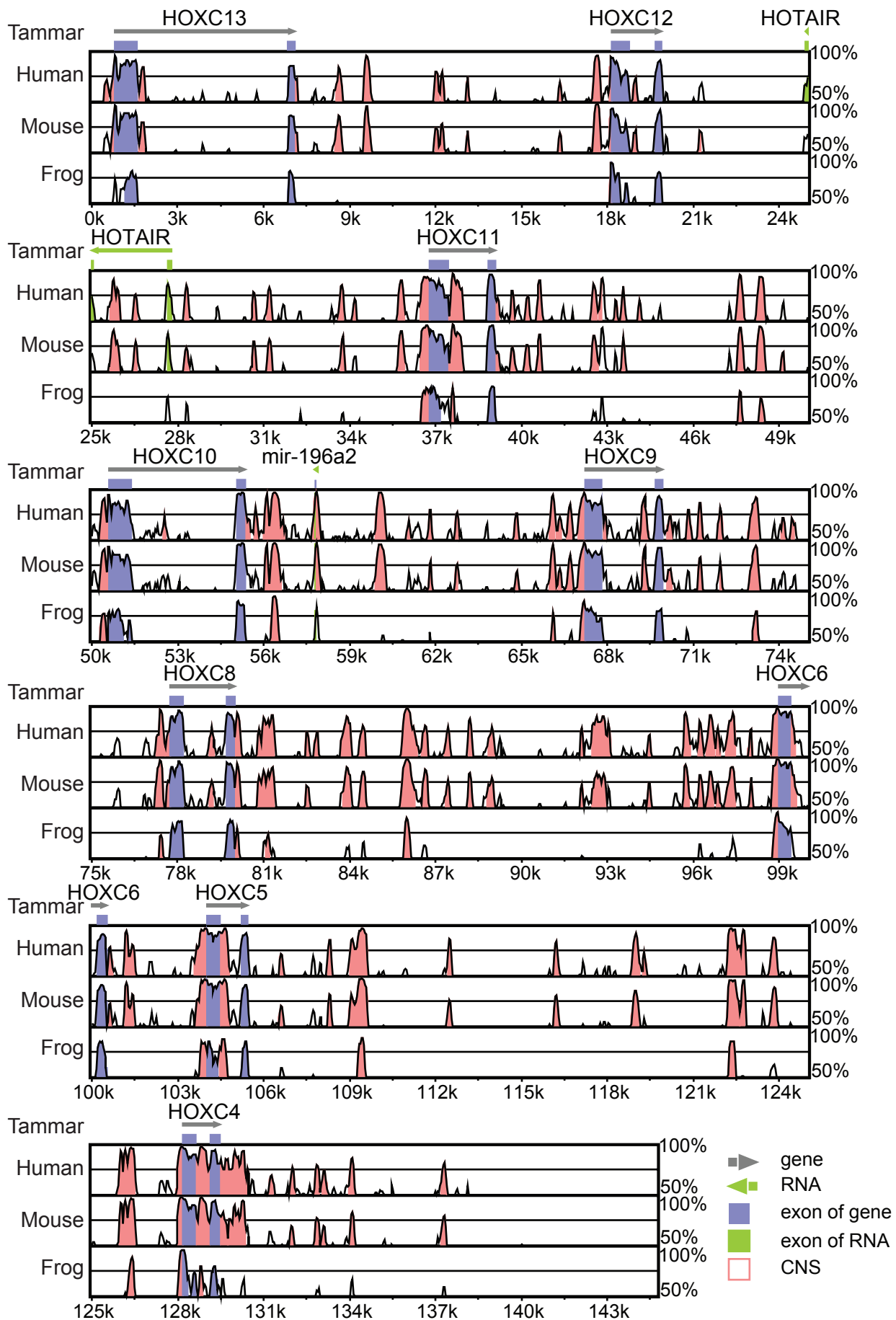

Supplement: Additional file 6 — Phylogenetic footprinting analyses ofHOXC cluster with mVISTA. mVISTA plot generated with HOXC genomic sequences from tammar, human (chr12: 52605461–52742874), mouse (chr15: chr15:102750000–102892969) and frog (scaffold_226: 269568–557892) with tammar as a reference. The information of long ncRNAs and microRNAs is same as in Figure 3. Other details as in figure Additional file 4. [file 1471-2164-13-251-S6.pdf]

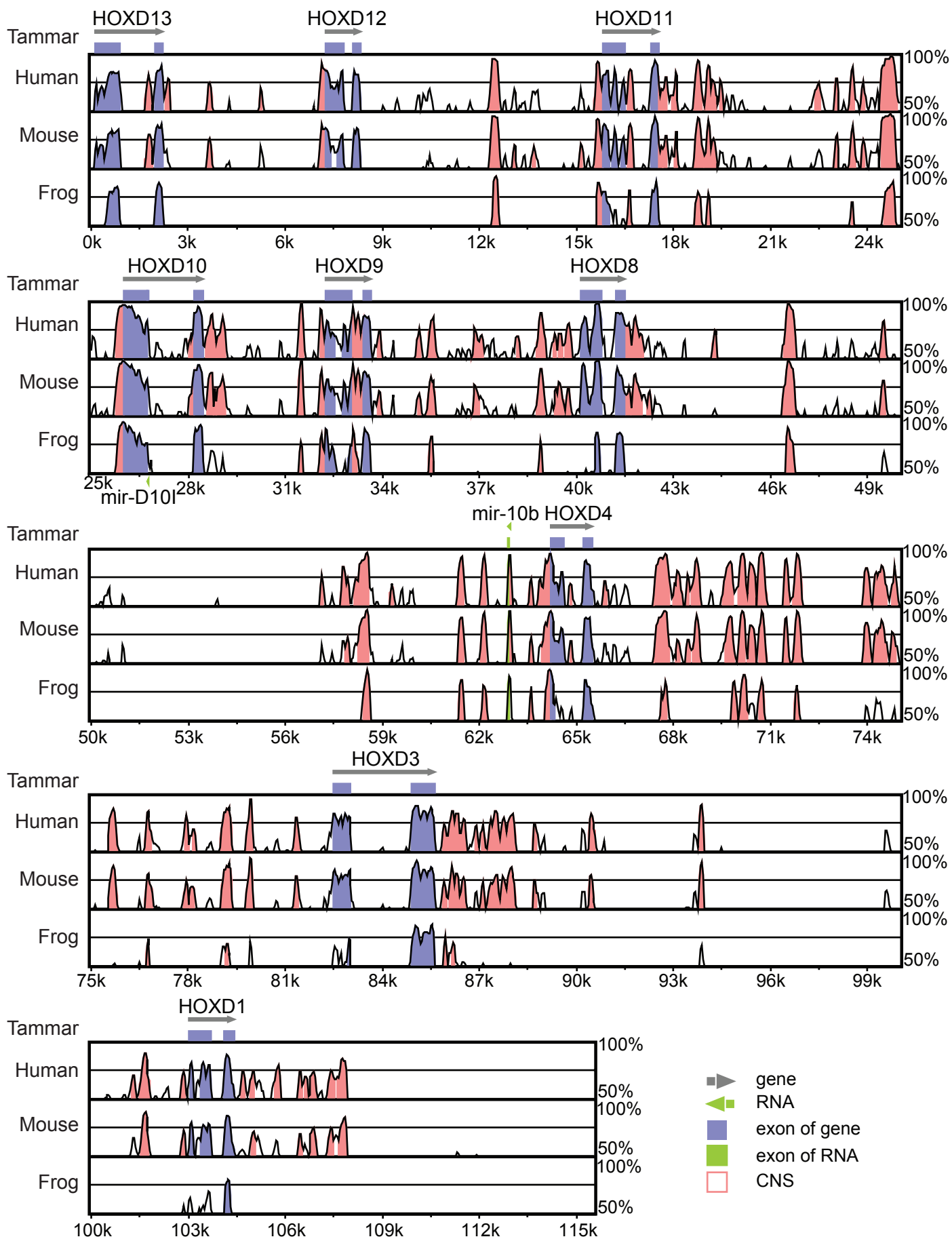

Supplement: Additional file 7 — Phylogenetic footprinting analyses ofHOXD cluster with mVISTA. mVISTA plot generated with HOXD genomic sequences from tammar, human (chr2: 176656359–176768195), mouse (chr2: 74497085–74613489) and frog (scaffold_163: 534804–660354) with tammar as a reference. Other details as in figure Additional file 4. [file 1471-2164-13-251-S7.pdf]

0.08

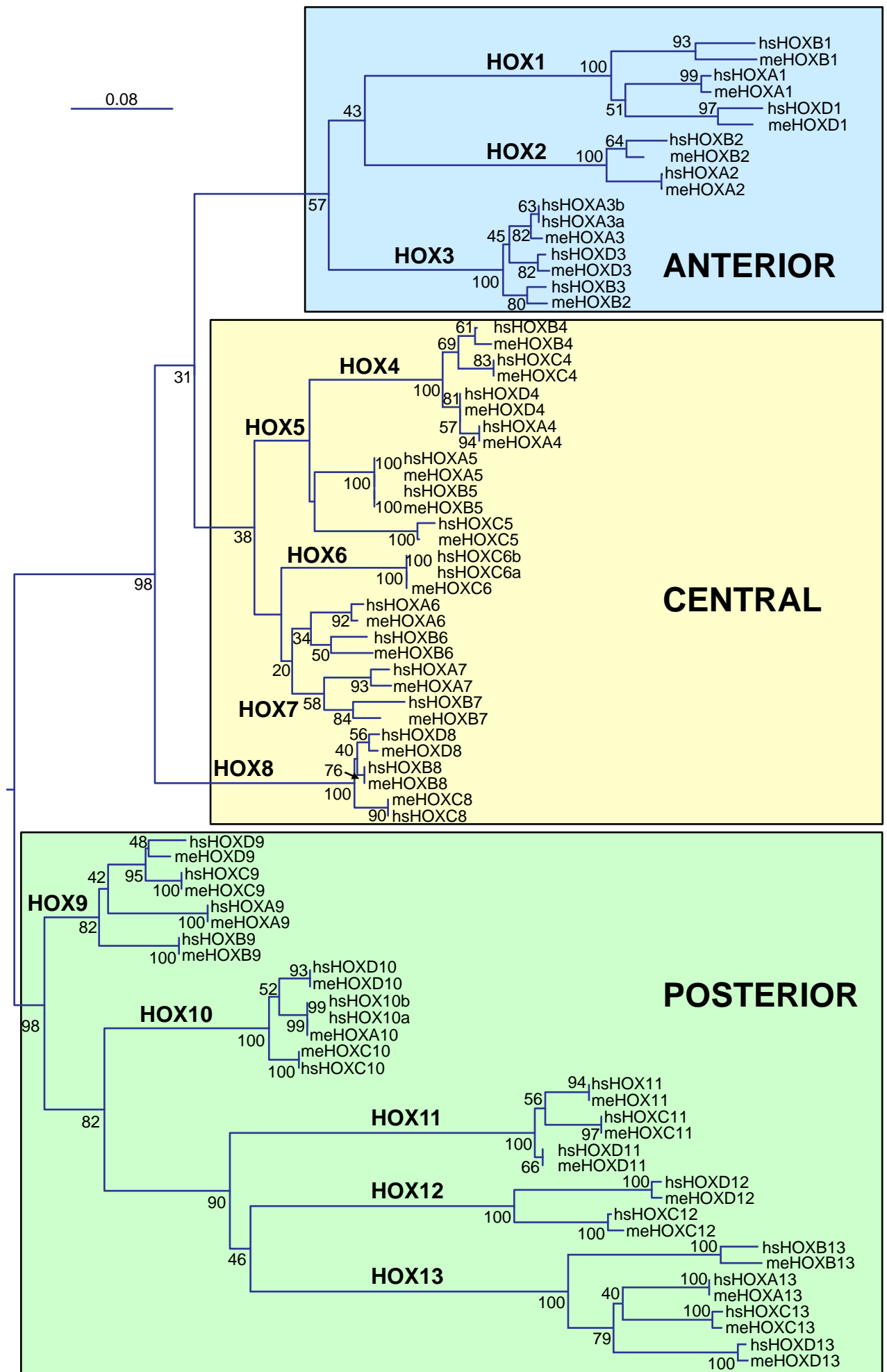

Supplement: Additional file 11 — Phylogenetic relationships and high-order grouping ofHOX families from tammar and human. A representative an unrooted tree with rooting that should be considered arbitrary. Phylogenetic analysis was based on the homeodomain regions with an extension of extra 20 amino acids on both sides from human and tammar. The phylogenetic tree was constructed using neighbor-joining method with 100 bootstrap replicates showing bootstrap support values on the nodes. 13 monophyletic groups were shown to form HOX1 to HOX13. Three big branches according to their functions during the developmental events are shown: anterior, central and posterior. [file 1471-2164-13-251-S11.pdf]
