# Supplementary material for: Evolution of coding and non-coding genes in HOX clusters of a marsupial
Source: BMC Genomics. 2012 Jun 18;13:251. doi: 10.1186/1471-2164-13-251 (PMC3541083; doi:10.1186/1471-2164-13-251)
Supplement: Additional file 8 — The sequences of lncRNAs and microRNAs in tammar. [file 1471-2164-13-251-S8.doc]

Additional file 8: The sequences of lncRNAs and microRNAs in tammar

1. **lncRNAs**

HOTAIR

>Exon4

GGGGGGAGGGCTTAGGGGGGAAAGGCCCCAAAGAGGCCAGTGTTTACAAGATCAGAAATGCCACGGCCATGTCCCTGGCCCAGAGAAAGCCCTGAAAATGGAGGACTAACGCCTTCCTTATAAG

>Exon5

AATAAACAGAGAATTAAGTGCTTTAGCCCAATGCTGCAATTACACTCAAG

>Exon6

CTTATCCTTTCCATCTTTATGATAAGTCTTGTTAACAGGAGTAGTGAGCCGGCTACATATATCTCTTCTGAGCCCACTCAGGCGTGGAGCTGGCGGATGTAGGTAACTACCAATGCACTAATTAATTGATTCCCTCAGATAGTAAAATA

HOTAIRM1

>Exon1

GAAAGTTTGCCTGTTCTTGCTGGTGATGGATCACCGTTTCAGTGGCATTTAAATCCCGGACGCTCCTCCATCAAGGTGACGCGCAGTCGCCCCCCCGGCCGCTGCAGCGGCGGCGG

>Exon2

CACACAGCCCAGCCCAGCCCAGCCCAGCCCAGCCCAGCCCAGCCCAGCCCTGCTCTCCGCTGGGCTCCCGAGGCCAAGTGAATTCTGCCCCTTCTTTCTCCCCCAGACGCCGCTGAAAGAGTTCTACGGGAGGGGGCCCGAGGAGGTGGGGGGCGGGGGGGCCACGTTCACCCCCTCGCAGGGCCCAGATTTCCTTTCTGGCCGCTAAAGCGATCGGTTAGTAAATCGGCTGAGCCCACTCACACATGTTGCTGGCCCTCTGTGTTTTCTCACG

>Exon3

GTCTGTTTGCCTGGGACCTCTCAGCAGCTGGGAGACTAATCAAACTCCTTTGGCTGGGTTTGACGGGACTTTTGTTACTGTCATCATTGGAATGTGGATGTTTTAAACAAAGGTGTAGAAATAAATGAATTGTTTGTGATTTAGCTATGGACCTAGAGACACATGGAGTTTTCTTTTTAAAGAAACTCCATATTCATCATGTAAAAGGGGAATATTTCATAGGCACTTTAAGTCTTCACTCTCAAAAACTCCAGCTTTCCCTGATGGAAAGGCTACACATTTCTCTTTCTTTTATGGGGTCACATCTATGGCTTGTACCTACTATAGTCTGGTAACAAAATCTAATCCACTTTAAAGGGAGGGGGGGAATGCTTCTCAGAAGCTCTGCCCATGCTCTCATTTTCCTTCTGTTGGCACCTAATCTTTGCAATAAAACTGAATGTTTCCCCCTAATAAATATGTTCAAATGGAAATAAAA

HOXA11AS

>Exon1

GCCATCTCAGGGGAAGCAACAGATCGGCACTTGGTATTCTCACCGAAAGCTCAGTAATCTCCAGTGTAACTCATGTTCGCTGGGGCACCCTCCGAGGACCGCCGGACGCGTGACAGACCTTGCCTTGCTCTCCGGGGGTGGGGGGGGGGTGGGAGAAGGGGGGCTCGAACCTCTTCCTCCCCCTAGACTACAGACCTCGACTGTCCGTTCCATCTCTCCCCTCTTTAACCTGGCTAGTGCCTGCTAGCCTTAGCCAAGGTGATATTGTGGAGGCATCGAGACTTGGGTTTGGGGCTGGGGGCCTTGTGGTTTCTGTCTCTGGTTTGGCCTCGGGCTCTCTCCTTCTCCCTGGCTTCCTGGACACCCCCTTCCAGCCCCCGTCAAGCTCTCCTGTTTCCGACTACCGCCCCACTCCCCCCCCCCCCCCGCCCCCGGCATCTCCCACCCCCGCGTCTATCTATTTCAGGGCTAGCCTCCGTCTCGGTGAATGTGCCGGGCTCGGGGCTTGTCTGATTTGCACGGTGGCTCCATTACACGCTCGCATTCACGGTCACTTCCGAGGCGCTCCAATAACTCCCCTCCCTTTACTAGAAGCCTCAGCAATCCCCCAAGGCGGTTTGCACCCGGGCTTTGCAGACCTGCTCAGTCAGGCAGGGCGTTCGTCTCACCCGGGATCTTTCTCGCTCTTCACCCCCACTTGCTCTTTCACCCCCCCCCCATCTTCCCACGCTTGCTCCCCAGCTTCGGAGCTCCCTGATGCAGAGATCTGGCCTAGGAGTCGGTTGGGGAAGTGAGGAAGGCTGCATGCTCTCCTCGATGCCTAGCACTGATGGCTTGGGGAACCAATGGCCATGAAGCAAGTCCCGGCTCCACAAACACTTCGAGTCTTGCTTAATTGCCAGCAGCCTAGTTAATGGGAATTGACATCCAAGGCTCTTTCCAGG

>Exon2

GAGGCCCAATCCAAGGGCACAAGGCTCGCTCAGCTCCGGGAGAGGGCCAGAGGACGTGAGAAGGACTGGCAAGAACATGGGCACATCAGGTCTCTTTTCCCAGGTTCTCCAAGCTTTCCCAGGTAAGGGAGAACCGAAGCTCTGACACTTGTCACGGCAATCTGGTCTCTGCAAGCTGTGTTTTTGCACAAGAGGAAAATAGCCAACCGAGGAGATCGATCTTATTTCTATCTCGCACTGAATTTATTTAATTTTACTGGTCAACACGGAGTTTCTTAAGAGTTTTATGTAAAGTGCCTAAAAATTCCTTCAGAGGAAGGAAAACAAAGCCAGGAAGTTCGGACGTTTGGATTTTCGTGCTTCTGATGAGCAAAGAGCGCAATTCTTTCTGACTCTTGTGTAAAAACTTCAAGCTCTCTATGGCTCTGGGAGGGAGGGTTGGCTCCTCCGGTTCCGTTGGATGCATTTTGGGGCGAAACCCATTTTTGAAGAAAAGTGAGGCAAGGGGAGGTGGAAAGAGGCCACATACAGGCATCCAAGTCACCTAAGACAGTAGTGATCCAAAGCAGACTTGGAAATGCGGAAGCAAAGAACGAGCCATGCTGGCTGGAAAAAACAAACCAAACCCTCGCCTTGGACTTGGCGGAGGATCCAGGCTGGGAGCTCGAGCTATTGTCCAGTTTCCCGGCCTCTCTGCACCGGTGGGCAGTGGGCTAGAGGAGTGCTGGGAGGTGGGTGAAACTGGGACGCC

1. **microRNAs**

>mir-196a1

UAGGUAGUUUCCUGUUGUUGGG

>mir-196a2

UAGGUAGUUUCAUGUUGUUGGG

>mir-196b

UAGGUAGUUUCCUGUUGUUGGG

>mir-10a

UACCCUGUAGAUCCGAAUUUGUG

>mir-10b

UACCCUGUAGAACCGAAUUUGTG

>meu-mir-6313

UUUGUUCUGUGAAGUUUGGAUU

> meu-mir-6313 precursor

CCUUUUGACUGAGUCUAAGUUUUACAGAACAAAUACUUUUAUUAGGGGGAUUUGUUCUGUGAAGUUUGGAUUCAGUCAAAAGG
