# Supplementary material for: Evolution of coding and non-coding genes in HOX clusters of a marsupial
Source: BMC Genomics. 2012 Jun 18;13:251. doi: 10.1186/1471-2164-13-251 (PMC3541083; doi:10.1186/1471-2164-13-251)
Supplement: Additional file 12 — Primers for RT-PCR. [file 1471-2164-13-251-S12.doc]

**Additional file 11:** primers for RT-PCR

|  | **Forward primer** | **Reverse Primer** | **Pro** | **Tem** |
| --- | --- | --- | --- | --- |
| ***HOXA1*** | GAGCACCCTATCCTCAACG | GCTGGAGTGGGAATAAGACAT | 249 | 57 |
| ***HOXA2*** | CTTTATCAATAGTCAGCCGTCG | GGAGGAGGAATCAGTGTCG | 123 | 51 |
| ***HOXA3*** | CTACTACGACAGTTCGGCAATC | TGGTGAGACAGAGGGAGGG | 394 | 57 |
| ***HOXA4*** | GGAGCTGGAGAAGGAGTT | CTTGGTGTTGGGCAGTTT | 157 | 53 |
| ***HOXA5*** | AGCCATACAGGGATTCAGC | CGATGTGCTCGTGGAGTTAG | 293 | 56 |
| ***HOXA6*** | TCCATTTTAACCGATACTT | TTTTCCTCTGTTTCTTCG | 169 | 49 |
| ***HOXA7*** | TCGCCAGACCTATACCCG | CAGCAGATCCCTCGTCCTT | 188 | 55 |
| ***HOXA9*** | GTCGGTCTTCGGCACTTC | CTTCGCTGGGCTGCTTAT | 317 | 57 |
| ***HOXA10*** | GGTCCTGAGCAAACGCAATG | GCCGAGACTGAAGCGAGCA | 642 | 61 |
| ***HOXA11*** | ATATTAACAAGGAGAAGCG | TTAGAGTAGCGGGTTTGC | 149 | 48 |
| ***HOXA13*** | AACGAGTGCCTTACACCA | TTGTCGCTCGGATAGATT | 123 | 48 |
| ***HOXB1*** | CCTCGTTATGGAAGTGGTC | TCAAAGGTTTGGGTGGTG | 404 | 51 |
| ***HOXB2*** | ACTTCAGGACGGAGGTGG | ATGCTGGTTCTGTCGTTTGT | 211 | 55 |
| ***HOXB3*** | CAGCACCAGTAGCAACAG | GGCACAGATAGCGATTGAA | 350 | 56 |
| ***HOXB4*** | TACCCAGCAACCACTCGC | TCCGCATCCAGGGATAGAC | 324 | 60 |
| ***HOXB5*** | CCGAAGAAGCAAGTCAGT | CTGGTAGCGAGTGTAAGC | 188 | 53 |
| ***HOXB6*** | GCCTCTTCTTCCTACTACCCA | GAACACTTCTGCTCCTCACTCT | 209 | 57 |
| ***HOXB7*** | CGTAAACGAGGCCGACAG | GGCAGGTGGTCTTGTTCT | 193 | 53 |
| ***HOXB8*** | TTAATCCCTATCTGACTCGC | TCTGCCTTCTGTCCTTCC | 223 | 53 |
| ***HOXB9*** | GGACGCTTAGCAGTTATTATG | ACGCTCTGGTGTTGGATA | 247 | 55 |
| ***HOXB13*** | ACCCGATGGCTGTTCCTA | GGTGGCGTTGGTCTTGAT | 229 | 56 |
| ***HOXC4*** | GTTTCATTACAACCGCTACCT | GTGGTCCTTCTTCCATTTCA | 124 | 52 |
| ***HOXC5*** | CACCGCCTGCTCCTTCTA | AGCCGCCTTCTGATTGTA | 164 | 55 |
| ***HOXC6*** | AATTTCACTTCAACCGTTACCT | TTCCCGCTTCTCCTCCTT | 203 | 56 |
| ***HOXC8*** | CCAAATACAAAGGCGGTGAG | GTGCGAGGCGTGCTGAAA | 131 | 56 |
| ***HOXC9*** | CGCCACTACGCCCTCAAA | TCGGGTCCAGTTCTGTCTTCT | 199 | 60 |
| ***HOXC10*** | CCTACCCGTCTTACCTTTCG | GGGCTGTCTCAGGACCACT | 180 | 55 |
| ***HOXC11*** | TGACAGACCGACAAGTGA | CAGTAGAGGGTTCCCAGAG | 101 | 48 |
| ***HOXC12*** | GCAAACCCTACTCAAAGC | CCTGCTGGTCACTAAGATTC | 120 | 52 |
| ***HOXC13*** | GGACAGTCAGGTGTATTGC | GTTCTGAGAGGTTGGTAGTG | 236 | 53 |
| ***HOXD1*** | ACAGGGCTCCGCATACGAC | TTGGCCGCTGAGAATGAAAG | 127 | 58 |
| ***HOXD3*** | TTTCAACCGCTACTTGTGCC | GGTGGTCGTACTCCTGTCCTG | 365 | 58 |
| ***HOXD4*** | CGTCGTCGGATCGAAATA | GTGTTGGGCAGTTTGTGG | 110 | 51 |
| ***HOXD8*** | ACGCTCTAGGACTCACC | CTGGCCCTCAGCTCTATC | 171 | 50 |
| ***HOXD9*** | ATCCACGCACGTTCCACC | TTTGACCTGCCTTTCTGT | 153 | 50 |
| ***HOXD10*** | CAAACACTGGAATTAGAAA | GAGAAGGTCAGATTAGCG | 191 | 49 |
| ***HOXD11*** | GCAAGCCCTCGTTCCTGTC | GTCCCTGTGCATCACCTCCT | 185 | 57 |
| ***HOXD12*** | CAGATCCGGGTATGTGGG | GCTAGGCTCGTTGGTGAAA | 346 | 57 |
| ***HOXD13*** | GTTCATCCTCTTCCTCCTC | CTTGAGTGCGTTCTGTTG | 221 | 56 |
| ***HOTAIR*** | AGGCCAGTGTTTACAAGATC | AGGCGTTAGTCCTCCATT | 81 | 50 |
| ***HOTAIRM1*** | TAAATCCCGGACGCTCCT | CAAACCCAGCCAAAGGAG | 308 | 59 |
| ***HOXA11AS*** | ATCGGCACTTGGTATTCTC | TAGCCCTGAAATAGATAGACG | 449 | 56 |
